# Gut Microbiota Modulation as a Novel Therapeutic Strategy in Cardiometabolic Diseases

**DOI:** 10.3390/foods11172575

**Published:** 2022-08-25

**Authors:** Yahkub Babatunde Mutalub, Monsurat Abdulwahab, Alkali Mohammed, Aishat Mutalib Yahkub, Sameer Badri AL-Mhanna, Wardah Yusof, Suk Peng Tang, Aida Hanum Ghulam Rasool, Siti Safiah Mokhtar

**Affiliations:** 1 Department of Pharmacology, School of Medical Sciences, Universiti Sains Malaysia, Kubang Kerian 16150, Kelantan, Malaysia or; 2 Department of Clinical Pharmacology, College of Medical Sciences, Abubakar Tafawa Balewa University, Bauchi 74027, Nigeria; 3 Department of Midwifery, College of Nursing Sciences, Abubakar Tafawa Balewa University Teaching Hospital, Bauchi 74027, Nigeria; 4 Department of Medicine, College of Medical Sciences, Abubakar Tafawa Balewa University, Bauchi 74027, Nigeria; 5 College of Medical Sciences, Abubakar Tafawa Balewa University, Bauchi 74027, Nigeria; 6 Department of Physiology, School of Medical Sciences, Universiti Sains Malaysia, Kubang Kerian 16150, Kelantan, Malaysia; 7 Department of Medical Microbiology and Parasitology, School of Medical Sciences, Universiti Sains Malaysia, Kubang Kerian 16150, Kelantan, Malaysia

**Keywords:** dysbiosis, cardiovascular disease, metabolic disease, probiotics, prebiotics, drug development, treatment, bacteria metabolite

## Abstract

The human gut harbors microbial ecology that is in a symbiotic relationship with its host and has a vital function in keeping host homeostasis. Inimical alterations in the composition of gut microbiota, known as gut dysbiosis, have been associated with cardiometabolic diseases. Studies have revealed the variation in gut microbiota composition in healthy individuals as compared to the composition of those with cardiometabolic diseases. Perturbation of host–microbial interaction attenuates physiological processes and may incite several cardiometabolic disease pathways. This imbalance contributes to cardiometabolic diseases via metabolism-independent and metabolite-dependent pathways. The aim of this review was to elucidate studies that have demonstrated the complex relationship between the intestinal microbiota as well as their metabolites and the development/progression of cardiometabolic diseases. Furthermore, we systematically itemized the potential therapeutic approaches for cardiometabolic diseases that target gut microbiota and/or their metabolites by following the pathophysiological pathways of disease development. These approaches include the use of diet, prebiotics, and probiotics. With the exposition of the link between gut microbiota and cardiometabolic diseases, the human gut microbiota therefore becomes a potential therapeutic target in the development of novel cardiometabolic agents.

## 1. Introduction

Cardiovascular diseases (CVD) are disorders of the heart and its associated blood vessels. They are most commonly coronary heart disease, cerebrovascular disease/stroke, aortic atherosclerosis, and peripheral vascular disease [1]. Cardiometabolic diseases (CMD) describe a span of conditions beginning with insulin resistance, progressing to metabolic syndrome and pre-diabetes, and ultimately end in CVD and type 2 diabetes (T2DM) [2]. CVD are leading causes of morbidity and mortality and result in huge global economic challenges [3]. The risk factors for CVD include unhealthy lifestyle practices (physical inactivity, alcohol, smoking, and diet), hypertension, diabetes, and dyslipidemia [4]. The unchanging escalation of risk factors for CVD, such as metabolic syndrome, diabetes, and obesity, mandates a probe for better approaches to prevent and modify the path of these CMD [5]. This led to significant interest in investigations on interactions between the gut microbiota and CMD [6,7,8,9].

Microbial sequencing analysis has supplied many facts about the existence of particular intestinal microbiomes linked with CMD [6,7,8]. Many investigations have demonstrated that disturbance of the gut microbiome equilibrium may play a role in altering CVD susceptibility by influencing aspects of immune response as well as susceptibility to obesity, insulin resistance, atherosclerosis, and thrombosis [10]. The microbiota also communicate with distal host organs through complex pathways via intestinal-microbiota-generated metabolites. This has been shown to affect the relevant factors of CVD, ranging from inflammation, obesity, and insulin resistance to more direct processes such as atherosclerosis and thrombosis susceptibility, dyslipidaemia, hypertension, and heart failure (HF) [11]. The term microbiota refers to a colony of an indefinite number of micro-organisms, collectively known as the microbiome, hosted by the human body. Microbiota reside mainly in the gastrointestinal tract (GIT) and especially the colon, where they enjoy an anaerobic, nutrient-rich environment suitable for their growth and colonization [12]. They contribute to various host metabolic functions [13] and are also able to produce biologically active metabolites that can affect host–receptor stimulation, transmission, and immunomodulatory functions [14,15].

The gut microbiota can therefore be considered an endocrine organ, because in addition to releasing their own agents, they also metabolize food substances and host matters into hormone-like signals. These products affect normal body functioning and chronic diseases including cardiometabolic ones [16]. The gut microbiota, therefore, partake directly or indirectly in our health status.

The response to the currently available evidence-based treatment modalities for CMD target only a fraction of the putative pathophysiological pathways [17]. They are expensive, accompanied by many unwanted side effects, and suboptimal despite adherence [14,18]. This is evident by the fact that CVD still maintains its lead in the cause of mortality globally. Hence, a more affordable and effective preventive and treatment strategy is urgently required. In view of producing better therapeutic targets, several studies have demonstrated that gut microbiota are closely associated with the development of CMD. Therefore, they are expected to become an essential target for CMD [17]. This recent awareness of the contributive role of gut microbiota and their metabolites in CMD consequently presents a fresh therapeutic target for drug discovery [19].

Review articles have studied gut microbiota in relation to cardiovascular and metabolic diseases elsewhere [12,20,21]. The focus of those studies was on gut microbiota and their metabolites in relation to disease development. They paid little attention to how gut microbiota and/or their metabolites could be utilized as a therapeutic strategy. Those that discussed the utilization of gut microbiota in the therapeutic approach did so on a general note. The present review summarizes the complex interplay between gut microbiota, their metabolites, and their potential roles in the development of CMD. It differs from the previous studies by further elucidating the targeting of the gut microbiome and its metabolites as a potential therapy or prophylaxis for CMD along the microbiota-related pathophysiological pathways of CMD development.

## 2. Gut Microbiota

Microbiota are described as a collection of microorganisms that share human body space. These organisms include commensals, symbiotics, and pathogenics [22]. The gut microbiota embodies bacteria, archaea, fungi, eukaryotes, and viruses or phages [6]. The genome of these organisms is referred to as the “microbiome” [23]. The continuous exposure of humans to the environment following birth aids the speedy colonization of the gut by 10 to 100 trillion bacteria that are usually non-harmful [24,25]. This figure is 10 times greater than the number of human cells and the component genetic information is about 100 times higher than the human genome [26,27,28]. The make-up of the gut microbiota in the healthy state is relatively stable and accounted for by the following major phyla: *Firmicutes*, *Bacteroidetes*, *Proteobacteria*, *Actinobacteria*, and *Verrucomicrobia* [25]. The first two phyla are the predominant ones, and other phyla include *Cyanobacteria* and *Fusobacteria* [12,25,26,27,28]. The microbiota that colonize the gut are mainly in the colon, where a nutrient-rich and anaerobic environment is provided [12]. The *Firmicutes*/*Bacteroidetes* ratio is viewed as a health indicator of intestinal microbiota [26]. The development of bacterial genome analyses has transformed microbial studies [10] by permitting the recognition of microorganisms and their relative abundance in the absence of traditional microbial culturing [29]. The commonly employed sequencing techniques are 16S analysis and metagenomic sequencing [10]. A better understanding of the role of the gut microbiota is therefore available since the microbiome analysis identifies and describes the gut microbiota by evaluating and categorizing their genomes and the corresponding metabolites [30]. This technology allows the investigation of the fundamental connection between the precise gut microbiome make-up and CMD [6,7,27,31].

Imbalance in the intestinal microbiome is also known as gut dysbiosis. It is associated with several conditions including gastrointestinal disorders, asthma, allergies, central nervous system disorders, metabolic syndromes, cancers, and CVD [32,33].

### Physiologic Role of Gut Microbiota

The gut microbiota are involved in the physiologic functions of the host via participation in digestion, metabolism, barrier protection, and immunological processes [34]. The major function of the gut microbiota are their involvement in digestion and nutrient utilization. The gut microbiota manufacture short chain fatty acids (SCFAs) from dietary fiber digestion via the saccharolytic pathway, which is responsible for the majority of microbiota-generated SCFAs. They also break down protein via the proteolytic pathway, which also produces SCFAs and other metabolites such as ammonia, thiols, and various amines [12]. The SCFAs obtained from the digestion of complex carbohydrates perform several roles and are mainly butyrate, propionate, and acetate [34]. Butyrate is vital in glucose homeostasis and energy regulation [31]. Acetate helps in fat storage regulation, while propionate acts as a raw material for gluconeogenesis in the liver [35,36]. These SCFAs can be interchanged by the microbiota based on a particular SCFA demand [37].

The three SCFAs vary in their physiological effect on the host [38]. Butyrate and propionate act as histone deacetylase (HDAC) inhibitors [39]. Inhibition of this enzyme attenuates hyperglycemia via regulation of glucose-6-phosphate expression and the ensuing gluconeogenesis [40]. This action was found to be responsible for the hypoglycemic effect of butyrate in a diabetic model [41]. Butyrate also activates the liberation of glucagon-like peptide 1 (GLP-1) from gut cells [42,43]. GLP-1 is capable of inciting pancreatic β-cell regeneration and apoptosis reduction in addition to inducing the synthesis and release of insulin [44]. Furthermore, butyrate promotes energy expenditure and stimulates mitochondrial function [45,46,47]. Butyrate has been demonstrated to induce satiety and decrease food intake by suppressing the activity of orexigenic neurons that express neuropeptide Y in the hypothalamus [48]. It also causes increased anorexigenic gut hormones [49,50].

Acetate, on the other hand, reduces the accumulation of fat via alteration of fatty acid oxidation [51] or synthesis and adenosine-monophosphate-activated protein kinase activity [52,53]. It decreases hepatic fat deposition by reducing circulating free fatty acids and de novo lipogenesis as well as improving mitochondrial efficiency [54]. In the adipose tissue, it increases mitochondrial activity and thermogenesis, therefore reducing fat accumulation even without appetite changes [55,56]. The benefits of SCFAs on glucose and energy homeostasis may appear paradoxical [57]. Although propionate has been traditionally known as a substrate for hepatic gluconeogenesis [36], the favorable outcome of propionate on glucose and energy homeostasis has been proven to be via intestinal gluconeogenesis (IGN). Propionate in the intestine is transformed into glucose (before it arrives at the liver) through IGN [57]. This IGN-produced glucose is sensed by the portal vein glucose sensor and can stimulate the hypothalamic nuclei concerned with the control of food intake with a subsequent reduction in food consumption [58].

The gut microbiota also participate in the production of essential amino acids, folic acid, vitamin B12, vitamin K, thiamine, and riboflavin through the breakdown of the residual substrates [59,60]. Other physiological roles of gut microbiota aside from digestive function include control of intestinal mucosal barriers, regulation of nutrient metabolism and utilization, activation of the immune system, and protection from pathogens, as well as interruption of pathogen transfer [61,62,63,64,65].

The intestinal microbiota, following the stimulation of pattern recognition receptor (PRR), also incite the formation of isolated lymphoid follicles in the bowel [66,67]. PRRs recognize pathogen-associated molecular patterns (PAMPs) on microbes to launch an immune response and are expressed on immunological cells such as dendritic cells and macrophages [28,66]. The gut microbiota, just like pathogenic organisms, also have PAMPs that are recognized by different classes of PRR, such as Toll-like receptors (TLRs) [34]. Microbiota also participate in host immune function by stimulating the maturation of gut-associated lymphoid tissues (GALTs). GALTs line the intestinal mucosa and form an essential mechanism of defense against pathogens [12,66,67]. The gut microbiota is involved in the activation and differentiation of various T and B lymphocytes [28,66]. In addition, the gut microbiota modulate the mucosal production of immunoglobulins (especially immunoglobulin A), which play a critical role in maintaining intestinal barrier function [28,66,68]. The intestinal barrier’s regulation acts in conjunction with the host immune system to prevent inappropriate inflammation [12,69].

**Figure 1:**
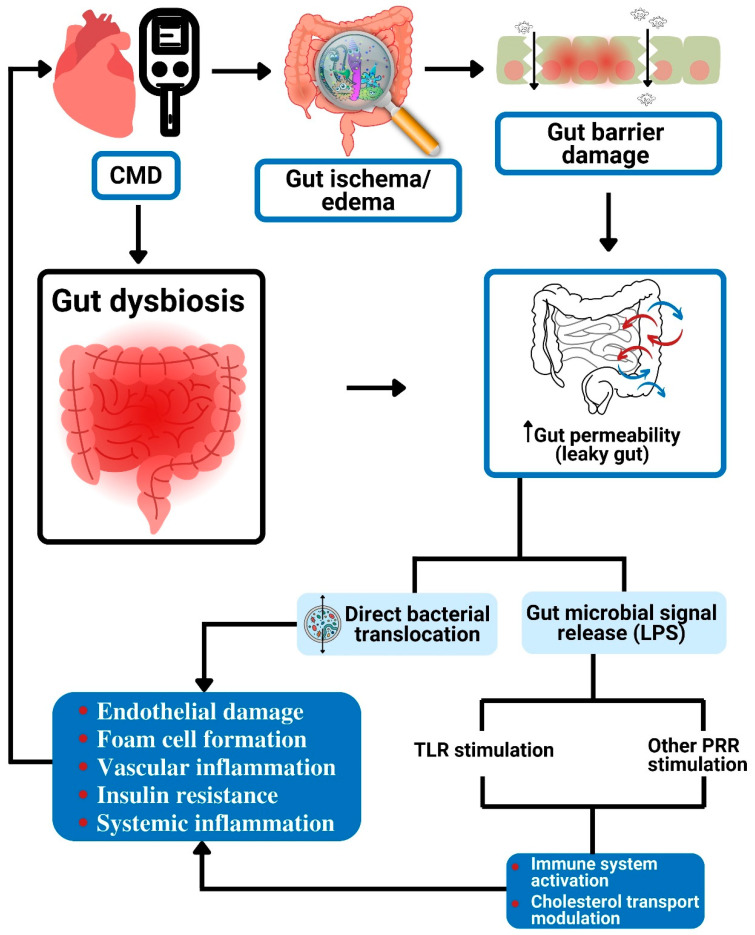
Gut dysbiosis and potential metabolism-independent pathways linked to cardiometabolic diseases. The increased gut permeability effect of gut dysbiosis causes direct bacterial translocation as well as the release of LPS into the blood circulation. The circulating bacteria result in vascular endothelial damage, formation of foam cells, inflammation, and insulin resistance. The released LPS stimulates TLR and other PRRs to cause immune reaction and modulation of cholesterol transport. All these can lead to CMD. CMD: cardiometabolic diseases; LPS: lipopolysaccharide; TLR: Toll-like receptor; PRR: pattern recognition receptor.

**Figure 2:**
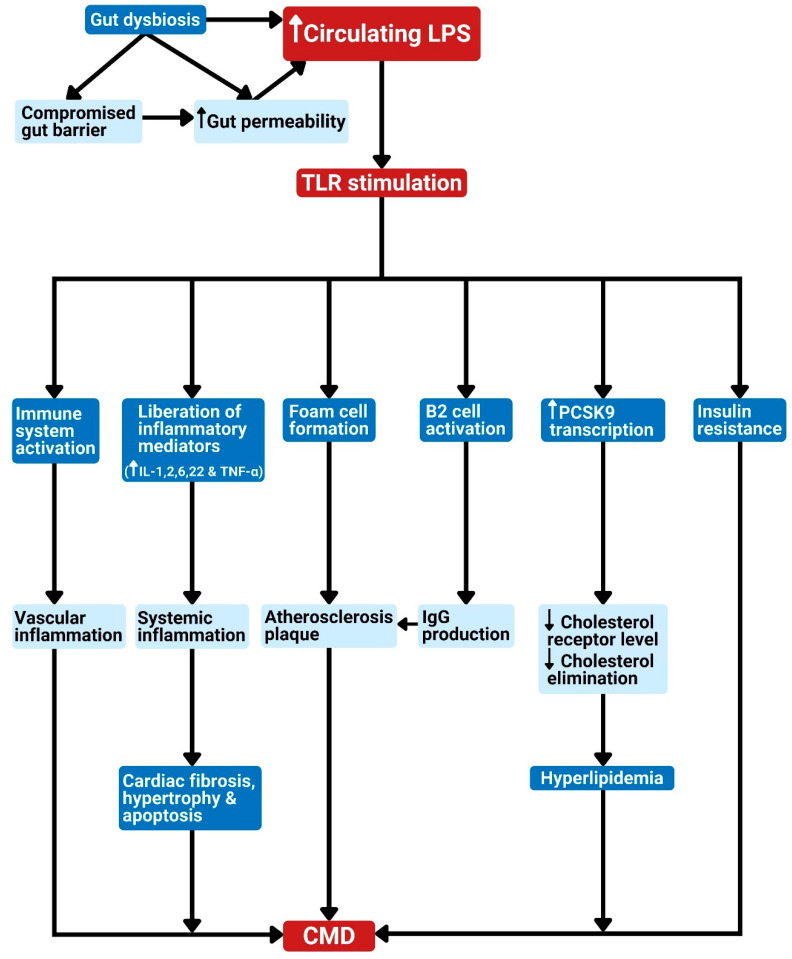
Gut dysbiosis and metabolism-independent bacteria signal release as linked to cardiometabolic diseases. Dysbiosis results in elevated bacterial LPS in circulation. The LPS in the bloodstream can stimulate TLR to produce the following effects: 1. Activation of the host immune system leads to vascular inflammation. 2. Liberation of inflammatory cytokines leads to systemic inflammation, which can cause cardiac apoptosis, fibrosis, or hypertrophy. 3. Promotion of foam cell formation leads to atherosclerosis plaque. 4. Stimulation of B2 cell activation in the spleen results in IgG production and eventually atherosclerosis development. 5. Stimulation of PCSK9 transcription, which reduces cholesterol elimination, leading to hypercholesterolemia. 6. The promotion of insulin resistance. These effects can lead to CMD. CMD: cardiometabolic diseases; LPS: lipopolysaccharide; TLR: Toll-like receptor; PRR: pattern recognition receptor; IL: interleukin; TNF-α: tumor necrosis factor-alpha; PCSK9: proprotein convertase subtilisin/kexin type 9; IgG: immunoglobulin G.

**Figure 3:**
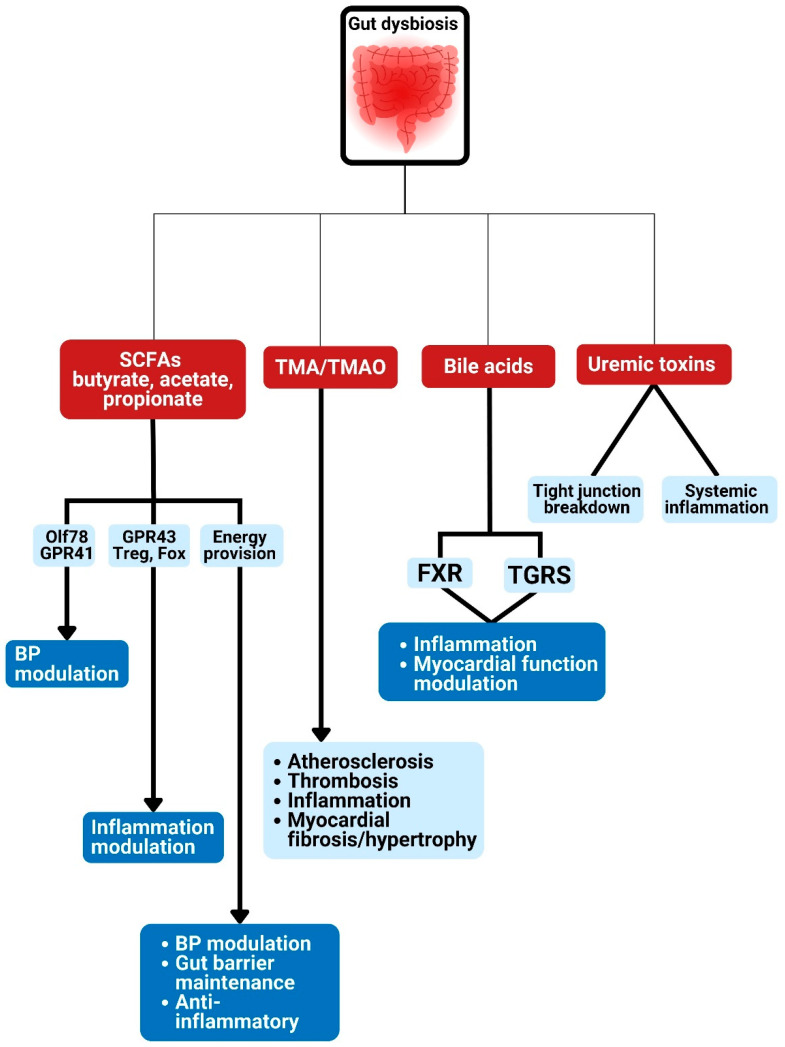
Gut dysbiosis and potential metabolism-dependent pathways linked to cardiometabolic diseases. Gut microbiota are linked to various CMD developments via the production of metabolites such as bile acids, short-chain fatty acids, trimethylamine-*N*-oxide production, and uremic toxin. SCFA: short chain fatty acids; TMA: trimethylamine; TMAO: trimethylamine *N*-oxide; GPR: G-protein–coupled receptor; Olfr78: olfactory receptors; BP: blood pressure; FXR: farnesoid X receptor; TGRS, G-protein-coupled bile acid receptor.

**Figure 4:**
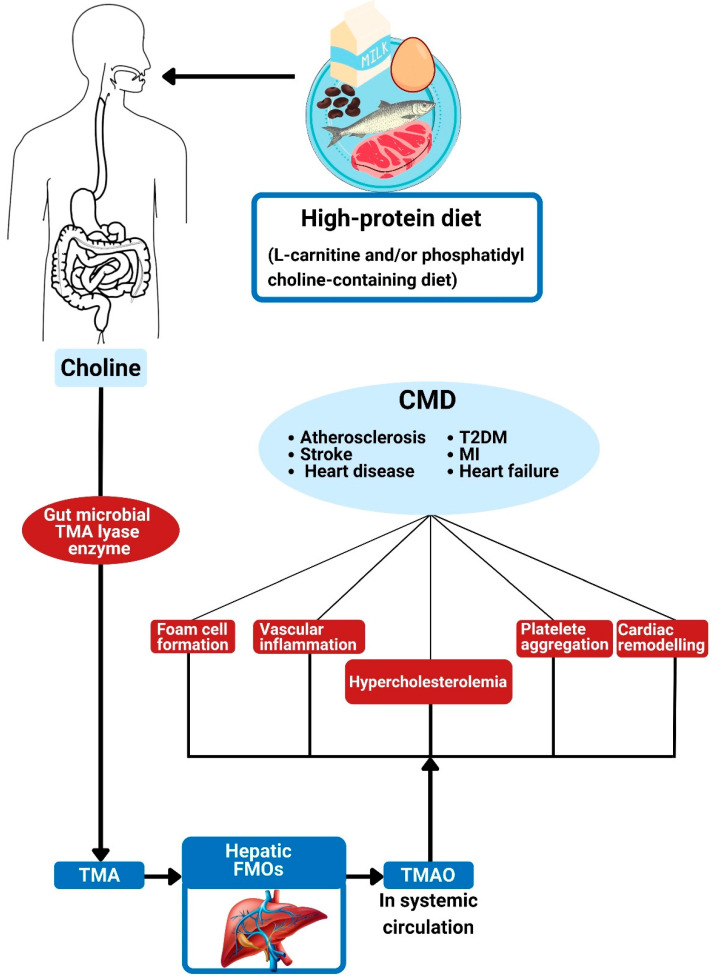
Trimethylamine *N*-oxide and its association with cardiometabolic diseases. The action of gut bacterial TMA lyase on dietary choline generates TMA. Hepatic FMOs convert TMA into trimethylamine *N*-oxide (TMAO). The effects of TMAO are associated with foam cell formation, alterations in cholesterol metabolism, platelet hyper-responsiveness, vascular inflammation, and adverse cardiac remodeling, all of which can contribute to CMD. TMA: trimethylamine; TMAO: trimethylamine *N*-oxide; FMO: flavin-containing monooxygenases; CMD: cardiometabolic diseases; MI: myocardial infarction; T2DM: type 2 diabetes mellitus.

Gut bacteria can also change bile acid composition and affect host metabolism. They produce secondary bile acids from unabsorbed primary bile acids. The secondary bile acid finds its way into the host blood system, where it modifies the pathways that are responsible for metabolism, inflammation, and energy balance [70]. Gut microbiota operates as a pseudo-organ with unparalleled endocrine potential. They produce plenty of regulatory chemicals via metabolism-independent (Figure 1 and Figure 2) or metabolism-dependent pathways (Figure 3), in comparison with the host endocrine organs that liberate a few hormones. These microbiota-derived products produce varying biological effects in the host [16] (Figure 1, Figure 2, Figure 3 and Figure 4). For example, microbial molecules capable of impacting host and disease states include neurotransmitters [71], uremic toxins [72], lipopolysaccharides (LPSs) [73], bile acids [74], nitric oxide [75], trimethylamine-*N*-oxide (TMAO) [76], gut hormones [77], SCFAs [78], vitamin B complex [79], vitamin K [80], GLP-1, and peptide YY (PYY) [81].

## 3. Gut Dysbiosis and the Disease States

Dysbiosis describes a situation of imbalance in the microbial ecology of the human system [23]. Gut dysbiosis refers to quantitative and qualitative alterations in the composition of the gut microbiota [30] and can result from exposure to several factors such as diet, stress (physical and psychological), inflammation, and antibiotic usage [82]. The use of antibiotics is the leading cause of dysbiosis and its potential to cause it depends on the spectrum of activity, pharmacokinetics, dosage, and duration of administration [83]. A broader spectrum antibiotic that is poorly absorbed with a large dosage and long period of administration will cause a more impactful dysbiosis. Stress may cause dysbiosis via the norepinephrine-induced growth of Gram-negative microorganisms or by stress-induced alterations in intestinal secretions and motility. Dysbiosis may result from the consumption of foods rich in sulfur compounds, high in protein, and/or high in meat via alterations in microbiota composition and/or metabolites [83]. Dysbiosis has been implicated in the pathogenesis of many disorders, which include CVD and metabolic diseases such as obesity and diabetes. It has also been associated with cancer and infectious diseases [35,84,85,86,87,88,89,90]. CVD has been linked to changes in the gut microbiota and/or its metabolites [91,92,93]. Special attention has been given in recent times to the interaction between CVDs (such as atherosclerosis, hypertension, and HF) and gut dysbiosis [94,95,96,97].

The dysbiosis model varies in various disease states. A remarkable elevation in *Lactobacillales* (Firmicutes) and a decrease in *Bacteroidetes* were reported in coronary artery disease (CAD) individuals [98], whereas diabetic individuals manifest a reduction in *Firmicutes* in addition to a non-significant increase in *Bacteroidetes* and *Proteobacteria* [99]. The proportion of *Bacteroidetes* to *Firmicutes* is a pointer to gut microbial health since these two account for the majority of gut bacteria [23]. Studies therefore propose that changes in the ratio of the microbe communities *Firmicutes* (F) and *Bacteroidetes* (B), known as the F/B ratio, can be possibly employed as a biosignature for disease conditions [100,101]. Available data have associated the actions and composition of the gut microbiome with health and disease [14]. Furthermore, the heightened presence of disease-causing organisms (such as *Bacteroides caccae* and *Escherichia coli*) and a depleted number of ”good” bacteria (such as *Eubacterium rectale* and *Roseburia*) have also been linked to higher risks of CVD [102,103].

In terms of specific bacteria that are directly associated with various CMDs, hypertension is associated with depletion of *Lactobacillus murinus* and *Anaerostipes* but increased *Prevotella*, *Erwinia*, and *Corynebacteriaceae* [9,104]. HF is associated with increased abundance of *Campylobacter*, *Shigella*, and *Yersinia enterocolitica* as well as a decreased abundance of *F. prausnitzii*, *Oscillibacter* sp., and *Sutterella wadsworthensis* in human feces [9,97,105]. Lower concentrations of *Roseburia intestinalis* and *Faecalibacterium prausnitzii*, as well as higher concentrations of *Lactobacillus* spp. and *Streptococcus* spp., have been observed in atherosclerotic CVD and diabetes [106,107]. Other bacteria found in atherosclerotic plaque include *Chryseomonas*, *Collinsella*, and *Veillonella species* [6,108,109]. Studies have demonstrated that obesity and diabetes are associated with lower *Akkermansia muciniphila* [110,111]. *Clostridium* spp. level is inversely related to diabetes and hyperlipidemia while *Lactobacillus* spp. is directly related to diabetes [112,113].

## 4. Pathophysiological Process of Gut Microbiota in Cardiometabolic Diseases

The gut microbiota interact with the host and modulate cardiometabolic processes to cause CMD via two main pathways [16]: metabolism-independent routes (Figure 1 and Figure 2) and metabolism-dependent routes (Figure 3).

### 4.1. Metabolism-Independent Pathway

Metabolism-independent routes involve the translocation of bacteria and/or their structural components into the circulation with inflammatory activation [73]. For example, peptidoglycan and structural components of the microbiota such as LPSs, both of which are constituents of microbial anatomy, can cause direct modulation of the host gut cells through Toll-like receptors (TLRs) [16,114].

#### 4.1.1. Direct Translocation of Bacteria

Studies have demonstrated evidence of the transfer of intestinal bacteria to the heart, with subsequent development of CVD and detection of intestinal bacterial genes in atherosclerotic plaques [115,116]. This shows that direct intestinal bacteria transfer is a mechanism through which gut microbiota lead to the development and progression of CVD [9].

#### 4.1.2. Releasing Gut Microbial Signals

Gut dysbiosis may result in higher gut permeability [9]. Additionally, splanchnic congestion associated with some CVDs, such as HF, causes intestinal edema, which compromises the intestinal barrier. The resultant effect of this is the leakage of bacterial components such as LPS into the circulation [23]. Higher gut permeability causes the release of bacterial LPS into circulation. The LPS in the bloodstream can stimulate Toll-like receptor 4 (TLR4), leading to a downstream signal that is transduced by myeloid differentiation primary response 88 (MYD88) to promote inflammation and foam cell formation [34]. The subsequent effect of TLR stimulation as it culminates in CMD is depicted in Figure 2.

Several studies affirm the association between inflammatory processes and CVD risk [117,118]. Atherosclerosis-associated CVD is now viewed as a chronic inflammatory disease leading to a series of biochemical and histological changes that culminate in plaque formation and rupture rather than just a cholesterol buildup event [119]. Patients with low-grade inflammation were found to be at risk of CVD despite having controlled cholesterol [120]. The CANTOS clinical trial (Canakinumab Anti-inflammatory Thrombosis Outcome Study) revealed that the risk of a cardiovascular (CV) event was significantly lowered following treatment with canakinumab, which is an inhibitor of interleukin (IL)-1β [121]. Moreover, IL-22 has been targeted for the treatment of CMD. It was observed to decrease inflammation and endotoxemia, protect the intestinal barrier, and enhance insulin sensitivity and endocrine actions, as well as control fat metabolism [122,123,124]. Better awareness of the impact of intestinal microbiota in initiating inflammation can provide treatment strategies for CVD [21]. The recognition of the gut microbiota responsible for immune reactions leading to CVD therefore serves as a therapeutic target for the prevention and treatment of inflammation-associated CVD. This makes immunomodulatory agents an optimistic therapy for CVD [21]. The limitation of this approach is the likelihood of opportunistic infections, especially in individuals having multi-morbidities, as usually noticed in CVD patients [21].

### 4.2. Metabolism-Dependent Pathway

Gut microbiota can directly affect CV risk factors and CVD development as well as progression. This direct effect is via the production of metabolites such as bile acids, short-chain fatty acids, trimethylamine-*N*-oxide, and uremic toxin (Figure 3) [16,80]. These metabolites can exercise systemic effects after gaining entrance into the circulation or local intestinal function. Many of these metabolites are immediately operative, while others require additional metabolism by the host to be functional [12,96,125,126,127,128].

#### 4.2.1. The Gut Microbiota Metabolite: SCFAs

SCFAs are 1- to 6-carbon products of gut microbial degradation of undigested dietary fibers and are predominantly acetate, propionate, and butyrate in the human body [19,23,129]. Besides being a macronutrient energy source supplying close to a tenth of host energy [130], SCFAs act as a modulator of several processes. These include inflammatory responses, autonomic systems, gluconeogenesis, blood pressure, and lipid metabolism, as well as other cellular functions [10,23]. Intestinal and systemic immune reactions are improved by SCFAs [131]. Numerous likely modulatory actions of gut microbiota on CVD are believed to be via SCFAs [10]. A considerable reduction in bacteria responsible for SCFA liberation has been discovered in patients with hypertension [132,133], HF [30], and other CVDs [7]. SCFAs thus appear to have a cardioprotective effect [30]. For example, gut microbial metabolites butyrate and propionate promote blood pressure reduction via modulation of the renin–angiotensin system [134,135]. SCFAs also modulate tight junction protein expression and preserve gut wall integrity [136]. Furthermore, they improve liver uptake of cholesterol and/or obstruct its production, therefore ameliorating hypercholesterolemia and ultimately preventing the development of CVD [137]. Butyrate prevents obesity [50] and CVD [138], enhances insulin sensitivity, and attenuates diabetes [47,139]. Furthermore, it decreases adipocyte and intestinal inflammation [140]. Butyrate-generating bacteria were appreciably fewer in feces of type 2 diabetic [106,141,142] and atherosclerotic patients in comparison with normal individuals [7]. Propionate acts via the G-protein-coupled receptor (GPR) 41 receptor to produce vasorelaxation and eventual hypotension in mice [134].

Dysbiosis associated with hypertension has been shown to be via changes in the bacteria metabolites, especially SCFAs [91]. Butyrate is mainly liberated by *Firmicutes*, whereas *Bacteroidetes* produce acetate and propionate [143]. The *Firmicutes* to *Bacteroidetes* ratio (the F/B ratio) in the feces can be a reader for an individual gut microbial metabolite [9]. Alterations in bacteria makeup that are linked to hypertension are accompanied by changes in the bacterial metabolite levels [91]. Hypertension animal models were associated with a significant reduction in bacteria liberating acetate and butyrate [132]. Hypertension research has also revealed greater F/B ratio in affected hosts [132,144,145].

SCFAs can act as signaling agents that control host function via receptor systems. The binding of SCFAs to GPR 41 or GPR43 can lead to the production of hormones such as GLP-1 in the intestine. This will modulate inflammation and energy regulation [146,147]. Circulating SCFAs can act through GPCRs to modulate blood pressure. The implicated GPCRs are in the resistance vessels on which their action results in modulation of tone and are mainly GPR41 and olfactory receptor 78 (Olfr78) [10,148]. Olfr78 is specifically pronounced in the juxtaglomerular apparatus of the kidney, where it controls the liberation of renin following stimulation by SCFAs [23]. GRP41 and Olfr78 are both pronounced in small resistance vessels, and they differentially influence vascular tone [23]. Stimulation of GRP41 may reduce blood pressure, as GRP41 knock-out mice are hypertensive [149], but activation of Olfr78 can increase blood pressure, as Olfr78 knock-out mice are hypotensive [134]. In addition, SCFA propionate binds to Olfr78 to elevate blood pressure after stimulation of renin release and may also bind to GPR41 to reduce blood pressure [150]. SCFAs are documented to modulate blood pressure in mice by regulating renin secretion via GPR41 and olfactory receptor 78 (Olf78) [151]. SCFAs bring about vasodilation and control liberation of renin and eventually blood pressure through GPCRs, majorly Gpr41 and Olfr78 [149,152,153]. The hypotensive effect of SCFAs on binding to GPR41 is via endothelium-dependent vasorelaxation [152]. SCFAs from gut microbiota also prevent oxidative stress and maintain the immune system [10]. Regulation of appetite via the gut–brain axis has been identified as another mechanism through which SCFAs affect metabolic processes [48,154,155]. In addition, SCFAs have an intestinal-barrier-protective function. Butyrate may encourage the proliferation of gut mucosa and restore injured gut epithelium, thereby preserving the integrity of the gut wall and decreasing gut and systemic inflammation [156]. The outcome of several animal investigations supports the modulatory effect of intestinal-microbiota-liberated SCFAs in CV processes. More studies are required to translate this effect into the management of human CVD [19].

#### 4.2.2. The Gut Microbiota Metabolite: Trimethyl Amine-*N*-oxide (TMAO)

Bacterial degradation of trimethylamine (TMA) [N(CH3)3] -containing nutrients such as choline, phosphatidylcholine, lecithin, and L-carnitine may liberate TMA following the action of a TMA lyase, which is a particular enzyme of intestinal microbes [125,128,157] (Figure 4).

The microbial choline TMA-lyase CutC and its corresponding activating protein CutD are recognized to break the C-N bond of choline to generate TMA [158]. Following absorption and subsequent transportation of intestinal TMA to the liver, the activity of hepatic flavin monooxygenase (FMOs; particularly FMO3) transforms it into TMAO (Figure 4) [159]. In addition to CutC/D, the release of TMA from other substrates is by the action of other enzymes that include carnitine monooxygenase (CntA/B) [160], CntA/B betaine reductase [161], TMAO reductase [162], and YeaW/X [163].

The main purpose of the conversion of TMA to TMAO by flavin-containing monooxygenase 3 (FMO3) is to enhance its solubility and elimination by the kidney [164,165]. The expressed effect of FMO3 on the intestinal absorption of cholesterol, reverse cholesterol transport (RCT), and bile acid formation in mice is possibly via TMAO, which hampers macrophage RCT, thereby elevating CVD risk by heightening plaque formation [166]. FMO3 was found to be upregulated and lead to elevated TMAO levels in obese-insulin-resistant mice [167].

The two primary determinants of circulating TMAO are high TMA-producing gut microbiota composition [168] and dietary consumption of TMAO substrates [17]. Dietary sources of TMA have such a large impact on the circulating quantity especially because TMA/TMAO production clearly relies on dietary derivations [23]. Dietary sources of TMA include red meat, milk, fish, eggs, soybeans, peanuts, and vegetables [169]. No-meat or white meat diets are correlated with appreciably lower quantities of blood TMAO compared to red-meat-rich diets [170]. Omnivores therefore have higher fecal TMA/TMAO and blood TMAO quantities than vegetarians and vegans [128].

TMAO is a risk factor for heart and kidney diseases [171]. It has several atherosclerosis and/or thrombosis and inflammatory actions in experimental animals [23]. TMA/TMAO generation causes the regulation of cholesterol and/or bile acid metabolism, including transportation [128]. Studies also demonstrated the regulatory role of TMAO in inflammatory responses, oxidative stress, and vascular dysfunction [127,172]. A strong association between high risk of CVD and circulating TMAO was demonstrated in germ-free mice and human studies [125,128,173,174,175]. Heightened TMAO quantities can predict higher risks of peripheral artery disease [176], CAD [177], myocardial infarction (MI) [178], stroke [173,175], and HF [179], even without taking cognizance of the traditional CV risk factors [173].

Elevated blood TMAO leading to foam cell formation and the subsequent atherosclerotic plaque was observed in normal gut microbiota mice following the feeding of a high choline diet [125]. Additionally, feeding a high choline diet to mice results in adverse myocardial fibrosis and ventricular remodeling [17].

Various microbial constitutions possess different capacities to liberate TMAO. It can therefore be theorized that a greater quantity of TMAO and elevated CVD risk will result from a higher TMA-generating dysbiotic bacterial constitution [173]. For example, *Prevotella* human gut bacteria is associated with more circulating TMAO compared to Bacteroides [128]. The variation in the TMA generating capability of these gut bacteria was linked to the differences in the presence of genes encoding proteins (particularly cutC and cutD) involved in choline utilization and TMA production [180]. The TMA-generating gut microbes are *Proteobacteria*, *Firmicutes*, and *Actinobacteria*. Others include *Prevotella*, *Akkermansia*, *Ruminococcus gnavus*, and *Sporobacter* [169]. These organisms are linked to atherosclerotic CAD [80].

High TMAO level is associated with hypertension [181], atherosclerosis [80,182], a higher risk of stroke [183], and death [80,182]. The hypertensive effect of elevated plasma TMAO is via its action on the renin–angiotensin system, which can also cause hypertension-associated CAD [171,184,185]. Elevated circulating TMAO impacts fat metabolism [76] mainly via decreased RCT as well as impaired cholesterol breakdown [34]. It also causes changes in bile constitution and transportation [125,128,186]. These encourage atherosclerosis development. Larger plasma TMAO increased CAD risk by 43% [76]. Endothelial dysfunction and C-reactive protein (CRP) are also linked to TMAO and associated with elevated LPS toxin [187]. TMAO may encourage monocyte adherence and increase vascular cell adhesion molecule-1, nuclear factor kappa B (NF-kB), and protein kinase C [188]. These cause stimulation of the inflammatory reaction and endothelial dysfunction, both of which promote CVD development [17].

Researchers have revealed that TMAO acts via calcium signaling to enhance platelet reactivity and the risk of thrombosis with a likely CAD. For instance, several mice thrombosis models demonstrated increased platelet activity and eventual clotting following intake of a choline-rich diet or gut-microbiota-associated TMAO surge as well as direct injection of TMAO [96]. Elevated blood TMAO causes circulating cholesterol buildup and eventual atherosclerotic plaque production by decreasing the expression of bile-acid-synthetic enzymes cytochrome-P450 (Cyp)7a1 and Cyp27a1 as well as exacerbating the macrophage scavenger receptor (ScR) CD36 [128]. ScR promotes the uptake of oxidized low-density lipoprotein (oxLDL), which encourages the generation of foam cells [189], while both RCT and cholesterol removal are decreased by reduced expression of the BA synthetic enzymes [190].

The compromised gut barrier and higher permeability associated with chronic HF also allow seamless passage of TMAO into the systemic circulation, resulting in heightened levels [17]. HF patients therefore have high TMAO and poor outcomes [174,179,191,192]. TMAO was noticed to cause myocardial fibrosis and cardiac hypertrophy in experimental rats [193]. Elevated levels of TMAO and cholesterol-associated foam cells as well as atherosclerotic plaque were observed in choline- or carnitine-rich-diet-fed mice [25,128]. Similarly, transplantation of high-TMA-generating microbiota transfers TMAO production as well as increased thrombosis risk into germ-free mice [96].

#### 4.2.3. The Gut Microbiota Metabolite: Bile Acids (BAs)

The gut microbiota influence the modulation of hepatic cholesterol metabolism [62,74], in addition to their role in the transformation of BAs, which affects circulating cholesterol levels [194]. Cholesterol is the major ingredient for BA synthesis, which is an important route of cholesterol elimination [190]. The role of BAs in atherosclerosis management is bidirectional. Cholesterol catabolism occurs via its synthetic process in addition to the athero-protective function of secondary BAs [195]. The modulatory effect of intestinal microbiota on BA metabolism is via the action of their enzyme bile-salt hydrolase (BSH), which transforms primary BAs into secondary BAs [35,195]. The atherosclerosis-promoting effects of gut dysbiosis may be due to the resultant reduction in BSH action of the new microbial components, leading to reduced cholesterol elimination and consequent atherosclerosis [34]. Primary BA synthesis occurs in the liver, from where it is secreted to emulsify fat in the intestine. Primary BA conversion to secondary BAs occurs in the intestine [35,190]. The intestinal lumen contains both primary and secondary BAs but more primary BAs. Most of the primary BAs are reabsorbed in the ileum (via the action of the BA transporter) and transported once again to the liver to be released once more [190]. Secondary BAs will probably be excreted through the feces since they are rarely absorbed due to their insolubility, therefore allowing the removal of cholesterol [56]. This whole process is termed enterohepatic circulation of BAs and is regulated by BA-signaling of the hepatic farnesoid X receptor (FXR) [190].

FXR activation by BA hinders the expression of cholesterol 7α-hydroxylase (CYP7A1) in the liver, thereby reducing the synthesis of primary bile acid [196]. Therefore, FXR activation by bile acids results in negative feedback modulating bile acid levels [19]. The microbial BSH is an important enzyme that modulates the stimulation of FXR and the ensuing signaling. Its role in the production of more excretable secondary BAs encourages fecal cholesterol removal [197]. Depletion of BSH as a result of dysbiosis makes more primary BA available for enterohepatic circulation re-absorption. This resultant exacerbated stimulation of hepatic FXR by excess BA suppresses the expression of the BA synthesizing enzyme CYP7A1 and the nuclear receptor LXR [195]. Increased suppression of CYP7A1 leads to reduced BA synthesis, which culminates in reduced uptake of cholesterol by the liver [34]. FXR-mediated LXR suppression leads to the reduction of cholesterol transporters ABCG5/G8 (which normally heighten the discharge of cholesterol from the liver and intestine) [195], encouraging cholesterol buildup in the enterocytes and hepatocytes. Decreased BSH action may cause atherosclerosis via cholesterol buildup and subsequent foam cell production [189].

Circulating BA quantities are linked with insulin resistance and diabetes mellitus [198,199]. The crucial place of intestinal microbiota in BA modulation was demonstrated when germ-free mice were found to have significantly larger amounts of BA in the enterohepatic circulation and no secondary BAs compared to normal mice [200]. In general, the gut microbiota regulate bile acid proportions. Dysbiotic and diseased conditions may result in increased primary bile acids, decreased secondary bile acids, stimulation of FXR, and reduced bile acid production, hence increase cholesterol and CAD development [80].

BAs are now acknowledged as signaling agents that influence cardiovascular activity [17]. This awareness was made conspicuous by the identification of bile acid receptors, such as the FXR and G-protein coupled bile acid receptor 1 (TGR5) [17]. FXR knockout mice and FXR agonists in the atherosclerosis model of mice predicted the athero-protective effect of FXR [201,202]. TGR5, which is chiefly stimulated by secondary bile acids, was associated with improved energy metabolism [203] and atherosclerosis prevention [204]. Stimulation of both FXR and TGR5 retract diet-associated atherosclerosis and metabolic disorders [205,206], but deficiency in both FXR and TGR5 promotes atherosclerosis via NF-κΒ stimulation [207]. FXR can enhance the perturbation of bile acid proportion and inhibit NF-kb, hence decreasing inflammation and enhancing myocardial function [208]. Microbiota metabolite bile acids, especially TGR5 agonists, produce a cardio-protective effect and enhance a myocardial reaction to stress [209]. Hence, TGR5 agonists and FXR can be novel targets for the treatment of CVD [17,19].

#### 4.2.4. Microbial Metabolite: Uremic Toxin

The gut microbiota urease can hydrolyze urea to form ammonia, which is later transformed into ammonium hydroxide. This process leads to the production of uremic toxins such as indoxyl sulfate and pcresyl sulfate. Negative CV outcomes were linked to indoxyl sulfate and pcresyl sulfate. Indoxyl sulfate was observed to directly act on cardiomyocytes by stimulating cardiac fibroblasts and collagen synthesis through activation of the p38 mitogen-activated protein kinase (MAPK), p42/44 MAPK, and NF-kB pathways, thus leading to adverse cardiac remodeling [210,211].

#### 4.2.5. Other Gut Microbial Metabolites

Apart from the earlier discussed gut microbiota-associated metabolites, other microbial metabolites with potential modulatory effects on the host are being recognized [19]. Tryptophan (Trp), phenylalanine (Phe), and tyrosine (Tyr) are aromatic amino acids that may modulate metabolic, immune, and neuronal responses. Trp is an essential amino acid and a precursor of serotonin, which is a neurotransmitter. The circulating microbe-generated metabolite of Trp was remarkably reduced in atherosclerotic patients [212]. Phe, another essential amino acid and precursor of Tyr, can be further transformed into neurotransmitters, norepinephrine, and adrenaline. Gut microbiota metabolites of Tyr and Phe were demonstrated to be related to the severity of MI in rats [213]. Further studies are required, based on the association between these aromatic amino acid metabolites and CVD. Furthermore, phenylacetylglutamine (PAGln), which is generated as dietary Phe is converted into phenylacetic acid, is also linked to CVD [214]. This gut-microbiota-derived metabolite acts to increase platelet activation and thrombosis risk via adrenergic receptors [215].

## 5. Gut Microbiota as a Target for Cardiometabolic Disease (CMD) Treatment and Prevention

The diverse relationship between CMD vulnerability and changes in gut microbiota make-up and metabolites has emphasized that gut microbiota is an unfamiliar modulator of CMD [9]. These connections are possible targets for new CMD therapy [9]. The host–microbiota interaction is made up of various levels at which potential therapeutic interventions can be instituted. These levels include dietary substrates, microbial ecology, and microbiota–host pathways that liberate metabolites that modulate host processes [21]. Agents that inhibit recognized gut microbial enzymes can also be produced [14]. The interesting part of this is that interventions directed at gut microbiota and/or their metabolism in lieu of the host may not necessarily be taken up into the host circulation, hence minimizing the likely adverse effects in comparison to those directed at host metabolism [19]. Among the challenges of therapeutically targeting the gut microbiota are the individual variations, in addition to differences, in gut microbiota make-up, which can affect the action of the medication. This may call for individualized treatment [14,19]. The gut-microbiota-directed therapeutic concept is based on targeting microbiota compositions, metabolic pathways, and mucosal barrier protection [30]. Our discussion of the intervention strategies in this review is based on these principles.

### 5.1. Targeting Whole Gut Microbiota

Gut microbiota, apart from acting as a medication target, may be used as a live treatment in the microbial intervention of management of disorders [19]. Live microbial therapy and/or whole gut microbiota target include fecal microbiota transplantation (FMT), single-strain microorganism or microbial consortia, and the use of antibiotics [9,19,21].

FMT is the process of direct transfer of healthy microbiota from an individual donor into the gut of a dysbiotic recipient with the aim of restoring the normal intestinal microbiota composition and function [216,217,218,219,220]. The recipient individual goes through a gut lavage of laxative therapy before undergoing FMT to improve the success rate of FMT [221]. FMT has been successfully employed for the treatment of Clostridium difficile infection and is lately gaining attention in the management of CMD [216,222,223,224]. A remarkable enhancement of peripheral and liver insulin sensitivity by 176% and 119%, respectively, was observed 6 weeks after fecal transfer from lean healthy individuals to metabolic syndrome patients and the observed enhancement was irrespective of weight differences [222]. This form of transplantation brought about a generalized intestinal microbial abundance, especially a higher proportion of butyrate-generating organisms [106,141]. Furthermore, FMT restored intestinal microbial equilibrium and prevented cardiac cell injury in a mouse myocarditis model [225].

Since the therapeutic application of fecal preparations was a practice of early Chinese [226], intestinal microbiota can be explored as a favorable derivation of live organisms for the treatment of CMD. Currently, the major drawback of FMT is a concomitant transfer of infectious organisms or endotoxins [9,12,227,228,229]. This could be bypassed by transplant of a specific category of bacteria rather than the whole fecal transfer [230,231]. The refinement of the CMD therapeutic role of FMT in terms of composition, route of administration, and dosage calls for additional research.

The application of live organisms to generally adjust microbial ecology in the management of CMD has received some great consideration. Probiotics are live microorganisms that provide their host with health benefits when used in the right quantity [232]. Only very few of them were endorsed as drugs, and the majority are being used as food supplements. They can be utilized therapeutically for varying cardiometabolic conditions. Remarkable reductions in blood lipid and/or glucose levels were observed following patient consumption of *Bifidobacteria-* and *Lactobacilli*-containing probiotics [233,234,235]. *L. acidophilus* ATCC 4358 treatment lessened atherosclerosis in ApoE^−/−^ mice [236]. Supplementation with probiotics also enhanced the metabolic profiles of diabetic patients [237].

Intake of *L. plantarum* by carotid atherosclerotic individuals increased bacterial diversity and affected intestinal SCFA generation [238]. Additionally, intake of *L. acidophilus*, *L. casei*, and *L. rhamnosus* each induced differential gene regulatory pathways in the human mucosa, as determined by transcriptome analysis. These response profiles were similar to those obtained for specific bioactive molecules and drugs, indicating the potential of gut microbiota used as naturally evolved drug candidates [239]. Obesity and type 2 diabetes (T2DM) were associated with reduced availability of intestinal *A. muciniphila* [110,240]. Improved metabolic profiles were therefore observed in obese and diabetic mice given *A. muciniphila* [241]. *Christensenella minuta* treatment was demonstrated to change the gut microbiota community and prevent obesity in mice [242]. *Lactobacillus rhamnosus* treatment decreased infarct dimensions and enhanced heart functions [243]. *Saccharonyces boulardii* was documented to enhance left ventricular ejection fraction in HF patients [244]. Other examples of probiotics are *Enterococcus*, *Bifidobacterium*, and *Streptococcus* [245]. Therapeutically administered probiotics to immunodeficient, debilitated patients may turn to opportunistic pathogens that can cause endocarditis [246]. This means that the probiotics approach in susceptible individuals should be used with caution.

Other research revealed contradictory effects in the use of probiotics on CVD and risk factors [12,116] probably due to preintervention microbial variability. For therapeutic purposes, an isolated microorganism or a specific category of microorganisms, known as consortia, can be established while trials in humans are conducted to establish their safety and efficacy.

Dietary habits also determine intestinal microbial heterogeneity. Acute dietary modification has resulted in commensurate alterations in the composition and quantity of gut microbiota [247]. Even though individual gut microbiota tend to be resilient, the abrupt nutritional alteration can adjust the microbial ecology [248,249,250,251]. Alterations in dietary sugar content were associated with alterations in *E. rectale* and *Roseburia* [252,253].

Some of the currently available antihypertensive drugs have been demonstrated to exert their action via gut microbiota modulation. For example, captopril, in addition to its inhibiting effect on the angiotensin-converting enzyme, also proliferates the gut bacteria *Allobaculum*. As a result of this bacteria, the stoppage of captopril treatment preserves the antihypertensive state [254]. Similarly, angiotensin II receptor blockers were demonstrated to maintain *Lactobacillus* levels, prevent gut dysbiosis, and restore the normal F/B ratio [255,256,257]. Moreover, statin drugs have been demonstrated to modulate gut microbiota. For instance, atorvastatin elevated *proteobacteria* levels and decreased *Firmicutes* levels in addition to normalization of dominant taxa in high-fat-diet-fed rats [258,259].

Pathogens are implicated in the pathophysiology of some CVDs, such as atherosclerosis [260,261,262,263]. Therefore, researchers have given attention to the use of antibiotics for the eradication of pathogen-associated CVD. For instance, decreased lipoprotein levels result from ampicillin therapy [264], while a reduction in systolic blood pressure was observed in spontaneously hypertensive rats following minocycline and vancomycin treatment [265]. Depletion of microorganisms and subsequently decreased plasma leptin and myocardial infarction ensued from oral vancomycin administration to rats [213,266]. Oral minocycline therapy modified hypertension after restoring gut microbiota balance and decreasing the F/B ratio [132,144]. This therapeutic approach is, however, limited by the restriction of effect to the period of antibiotic administration, therefore requiring chronic use, with its consequent likelihood of antibiotic resistance and depletion of beneficial bacteria [9,21,30]. There is presently no proof of the overall beneficial effect of vague antibiotic therapy in human CVD management. Antibiotics, therefore, appear to be more appropriate for the elimination of disease-causing microorganisms, instead of a prolonged prophylactic application.

Higher *Bacteroidetes* and lower *Firmicutes* were observed after 10 weeks of moderate to severe aerobic exercise by obese adults [267]. Similarly, regular exercise attenuates obesity development and causes changes in the gut microbiota composition in a mice obesity model [268,269]. Furthermore, exercise increased the percentage of *Bacteroidetes* and decreased the percentage of *Firmicutes* regardless of diet [270], and high-intensity interval training increased the gut *Bacteroidetes* to *Firmicutes* ratio during diet-induced obesity [271]. Rugby players were found to have a higher number and more diverse gut microbiota compared to their non-athletic counterparts of similar age and body mass index [272]. This study also revealed that exercise may increase the α-diversity of gut microbiota and the abundance of the bacterial genus *Akkermansia*. Voluntary exercise impacted the *Bacteroidetes*/*Firmicutes* balance and prevented diet-induced obesity, in addition to causing improved glucose tolerance [273].

A recent study has demonstrated a depletion of opportunistic pathogens and accumulation of intestinal-wall-protecting bacteria in association with improved lipid profile and insulin sensitivity after nutritional intervention with prebiotics and whole grains [274]. Anthocyanin-containing fruits such as blueberries could also heighten the diversity of gut microbiota [275]. Dietary fortification with magnesium acetate also modified hypertension after the restoration of intestinal microbiota balance and decreased the F/B ratio [132,144].

### 5.2. Treatment Targeting LPS/Strengthen Intestinal Barrier

Interventions leading to the reduction of circulating LPS have been explored for the management of CMD. Some of the interventions are listed below:

The high quantities of circulating LPS associated with certain CMD could be reduced through physical exercise [276,277]. This will subsequently ameliorate CMD. For instance, physical exercise led to changes in the structure of intestinal bacteria that favor reduced LPS and avert heart impairment in mice with myocardial infarction [278]. In rats fed a high-fat diet, both acute and chronic exercise may induce a significant decrease in the TLR4-mediated signaling pathway in the liver, muscle, and adipose tissue, accompanied by the concomittant reduction in serum LPS levels and improved insulin signaling and sensitivity in metabolic target tissues [279,280].

Decrease atherosclerosis of the aorta related to reduced plasma and fecal LPS as well as lower gut penetrability was observed following oral administration of *Akkermansia muciniphila* to Apolipoprotein E (ApoE)^−/−^ mice fed with a Western diet [281]. Similarly, reduced circulating LPS from *A muciniphila* treatment of metabolic syndrome patients improved their lipid profile and insulin resistance without alteration of body weight [282].

Antibiotics can reduce the fecal and circulating LPS. Rifaximin, tobramycin, and polymyxin B, for instance, could decrease gut bacterial translocation and decrease gut LPS, aside from their regular bacteriostatic and bactericidal action [283,284]. The limitations of antibiotic intervention were nonetheless mentioned earlier on.

### 5.3. Treatment Targeting Inflammation

Studies have associated inflammation with CMD [121]. The recognition of intestinal microbiota associated with immunological reactions involved in the pathogenesis of CMD can serve as a therapeutic target in inflammation-associated CMD [21]. Prebiotics are indigestible food materials that enhance the growth of beneficial gut microorganisms [17]. Inflammatory cell invasion in rats was significantly decreased with prebiotic oligofructose [285]. An appreciable alleviation of inflammation and hypertension was demonstrated in patients with hypertension following consumption of the dietary approach to stop hypertension (DASH) and the Mediterranean diet [286,287,288,289]. The application of immunotherapy may however be limited by a higher likelihood of opportunistic infections, especially in individuals with multimorbidity [17].

### 5.4. Treatment Targeting SCFAs

Several researchers including Gordon [121] have evaluated the role of SCFAs in CMD. The enhanced insulin sensitivity observed in metabolic syndrome patients after FMT from lean individuals was linked to higher butyrate-generating microorganisms and subsequent higher fecal SCFAs [222].

Supplementation with 1% butyrate halved aortic lesions in mice [290,291]. SCFAs also reduce hypertension in animals [135]. Another research revealed that treatment with *Roseburia intestinalis* (butyrate-liberating bacteria) attenuated atherosclerosis development via butyrate [146]. Lifestyle modification (including diet and exercise), which is presently a vital clinical intervention in the management of CMD, alters gut microbiota composition and function, including SCFA production [23].

Studies have demonstrated the attenuation of CMD by SCFAs liberated from gut microbiota degradation of prebiotic fibers [126,154,174]. Prebiotics are indigestible molecules that provide a beneficial effect to the host via alteration in the make-up and/or actions of the gut microbiota. They are usually in form of complex saccharides or oligosaccharides [292]. An energetic tool in the prevention and treatment of CMD is the regulation of gut microbiota through diet. For example, intestinal microbiota modulation via a high-fiber diet and acetate intake attenuated cardiac diseases and hypertension [144]. Similarly, modulation of intestinal microbiota with a high-fiber diet resulted in the growth of beneficial bacteria, heightened generation of SCFAs, and caused attenuation of elevated blood pressure [293]. A high abundance of acetate-liberating intestinal bacteria and subsequent remarkable reduction in fibrosis and hypertrophy of heart cells, as well as blood pressure and HF prevention, were demonstrated in mice fed a high-fiber diet [144]. Furthermore, hypertension reversal ensued through intake of butyrate- and acetate-generating corn and *Clostridium butyricum* by hypertensive rodents [294]. Additionally, oral intake of butyrate, propionate and acetate decreased insulin resistance and body weight in high-fat-diet-induced obese mice [46].

A high generation of SCFA can also result from the consumption of vegetables, legumes, and fruits [295]. The favorable role of a plant-based diet compared to animal-based ones has been attributed to their ability to modulate local and systemic SCFA generation [23]. A remarkable decrease in fecal butyrate and acetate level was demonstrated with the shifting of individuals from plant- to animal-based foods [251]. Propionate averts hypertension, vascular dysfunction, heart fibrosis, and hypertrophy [296]. A decrease in adverse cardiac remodeling and hypertension ensued when gut microbial metabolite (acetate) or a prebiotic (high-fiber diet) was administered in a rodent hypertension model [144]. Furthermore, carotid artery endothelial dysfunction was attenuated following administration of prebiotic inulin-like fructans to ApoE^−/−^ mice [297]. Prebiotic inulin treatment also remarkably reduced atherosclerotic lesions in ApoE^−/−^ mice [298]. A plant polysaccharide-rich diet increased butyrate generation by gut microbiota and attenuated atherosclerosis compared to low-plant polysaccharide diets [146]. Similarly, atherosclerosis was attenuated by microbiota of ApoE^−/−^ mice when fed a high-plant-polysaccharide-containing diet, as contrasted to a Western diet-fed one [299]. Augmentation of the diet with ginger also modifies gut microbial ecology and incites enhanced metabolism of fatty acids [170].

Studies have revealed the ability of probiotics to positively modulate fat metabolism [300,301]. The probable mechanisms of probiotics involve opposition of disease-causing organisms, the liberation of antimicrobial agents, and pH modification [302,303]. *Lactobacillus* sp. administration was linked with remarkable alteration in colonic SCFAs in carotid atherosclerotic patients [238]. Symbiotic formulations are dietary adjuncts with a blend of probiotics and prebiotics that can modify intestinal metabolism [294]. The commonly adjoined probiotics in symbiotic formulations are *Bifidobacteria*, *Lactobacilli*, *S. boulardii,* and *B. coagulans*, while the prebiotics are oligosaccharides, dietary fibers, and inulin [304].

FMT to metabolic syndrome patients from lean individuals was linked to a higher abundance of butyrate-generating bacteria (such as *Roseburia*) and resulted in improved insulin sensitivity [222].

Current studies demonstrate that exercise modifies the intestinal microbiota for its cardiovascular effects. Research revealed that it increases the ratio of *Firmicutes* to *Bacteroidetes* [305,306] as well as elevates the amount of the microbial product butyrate [307]. The positive effect of exercise on intestinal microbiota was interim and abated following the stoppage of the exercise [307]. This makes a longer period of exercise a necessity for remarkable long-lasting effects [9]. The gut metabolism of athletes is found to generate a high level of SCFA [308]. Voluntary running in animals is also associated with microbiota diversity and attendant elevated butyrate levels [309]. The butyrate may inhibit the activity of histone deacetylases and therefore influence immune modulation and decrease oxidative stress [310]. It can also regulate gut motility and barrier integrity as well as inflammation and visceral sensitivity [310,311]. All these partake in CMD modulation. Huang et al. [312] studied rats and demonstrated a pronounced antioxidant function and tricarboxylic acid cycle with endurance training. With regards to the use of exercise to modulate gut microbiota for CMD treatment, further research is needed to answer the questions about the types, timing, and conditions of exercise to achieve a remarkable impact on CMD.

Favorable effects of SCFAs have been demonstrated in hypertension models in rats. Local inoculation of acetate into the colon overturned hypertension in rats [313]. Oral administration of propionate led to enhanced vascular action, decreased blood pressure, and adverse cardiac events in hypertensive mice [296]. Butyrate or propionate administration prevents myocardial harm in hypertension models [135,296]. Sodium butyrate therapy in rodents resulted in improved insulin sensitivity and attenuation of obesity [314]. Tributyrin was synthesized to solve butyrate’s problem of offensive taste and odor. It is a prodrug that carries three molecules of butyrate esterified to glycerol and with comparable cardiometabolic action to and better pharmacokinetic parameters than butyrate [315]. Tributyrin treatment of obese mice diminished insulin resistance and inflammation [19]. Another research revealed that fat accumulation and atherosclerosis were remarkably attenuated with tributyrin treatment in ApoE^−/−^ mice. This is an optimistic approach for CVD prophylaxis [146].

### 5.5. Treatment Targeting TMA

Therapeutic approaches that are directed toward halting TMAO generation and elimination of TMAO and its progenitor (TMA) have gained recognition owing to the association of TMAO with cardiometabolic diseases by several studies, as mentioned earlier. Hence, targeting the gut microbiota for reduction of TMA generation will be a likely treatment strategy for CMD [19]. Apart from pharmacological agents, probiotics can be employed for the inhibition of metabolic pathways in gut microbes to reduce TMAO liberation [168].

Mediterranean diet consumption led to remarkably reduced TMAO and could attenuate CMD [316,317]. CMD can be prevented by limiting the intake of a choline/carnitine-rich diet since nutritional intake is the main source of TMAO [128]. Western diets are carnitine/choline-rich while vegetarian diets are carnitine-choline depleted [170,318]. A high-fiber diet decreased plasma TMAO [319]. Although there is no consensus on the effect of saturated fat and red meat intake on TMAO levels [170,320,321], individuals following a vegetarian diet regimen had reduced plasma and urinary TMAO [292,322]. It is therefore thought that this dietary approach could reduce TMAO generation and subsequently attenuate CMD [323].

It is postulated that the transfer of low TMAO-liberating microbiota to patients with heart failure or its risk factors could decrease TMAO, yet none of such has been demonstrated clinically [17].

Broad-spectrum antibiotic administration to individuals led to decreased intestinal microbiota and a remarkable reduction in TMAO levels [173,175]. The use of broad-spectrum antibiotics in elderly mice reduced plasma TMAO and subsequently attenuated endothelial dysfunction and aortic stiffening to a similar level as the young mice [324]. CVD prevention in humans using long-term antibiotics is not practicable due to the previously mentioned challenges of this approach [21].

Reduction in TMAO and altered platelet aggregation were observed in individuals with high TMAO that were treated with low-dose aspirin [96,318]. The low-dose aspirin can also change the gut microbial composition [325].

Targeting bacterial enzymes involved in TMA generation for inhibition are being investigated as a preventive or therapeutic approach for CMD [95,128,158,160]. For example, a structural analog of choline 3,3-dimethyl-1-butanol (DMB) is a model microbial TMA lyase inhibitor used for this purpose [326]. Reduction in TMA/TMAO ensued inhibition of TMA lyase and inhibition of TMA lyase modified CVD [327]. Foam cell production and subsequent atherosclerosis were impaired after oral administration of DMB, which resulted in decreased plasma TMAO in ApoE^−/−^mice on a choline-augmented diet [95]. Another study also demonstrated the anti-atherogenesis effect of DMB [328]. Furthermore, decreased ventricular remodeling and enhanced hemodynamic status followed DMB administration [329]. More potent TMA lyase inhibitors such as fluoromethylcholine, iodomethylcholine, chloromethylcholine, and bromomethylcholine have been invented. These compounds specifically act locally on the gut microbiota with minimal systemic exposure of the host, thereby limiting adverse effects [21,330]. TMA lyase inhibitors, including DMB, are harmless to the microbes. Therefore, they do not cause selective pressure like antibiotics do, and the risk of resistance is minimal [21].

Aside from TMA lyase (CutC/D), other enzymes involved in the generation of TMA from other precursors include CntA/B [160], betaine reductase [161], TMAO reductase [162], and YeaW/X [163]. Clinical trials have demonstrated that dietary indoles of Brussels sprouts inhibited FMO3 and thereby prevented the conversion of TMA to TMAO [331]. Drug development that targets a reduction of TMAO production via inhibition of TMA lyase is preferred to specific FMO3 inhibition because elevated plasma levels of TMA result in a condition termed trimethylaminuria, which brings about an offensive “fishy” odor [332]. Another enzyme inhibitor (meldonium), which is an analog of carnitine, produces an anti-atherosclerotic effect by competitively inhibiting microbial carnitine palmitoyltransferase-1 (CPT1), resulting in the attenuation of microbial TMA generation [333]. Furthermore, phospholipase D (PLD) is one more intestinal microbial enzyme that can be targeted for drug development. PLD is involved in TMA generation by liberating free choline from phosphatidylcholine, the predominant dietary form of choline. The good news is that gut microbial PLD can be preferentially inhibited without affecting the host enzyme, owing to the phylogenetic differences between host and microbial PLD enzymes [334]. Therefore, future therapeutic action with innocuous enzyme inhibitors directed at gut microbiota presents an innovative strategy for the prevention and treatment of CMD. This nonetheless requires clinical studies for validation [12,21].

### 5.6. Treatment Targeting Bile Acids

A secondary bile acid, ursodeoxycholic acid (UDCA), was studied as an innovative treatment for rodent obesity [335]. An FXR agonist and semisynthetic analog of bile acid, obeticholic was thought to have the ability to decrease bacterial translocation and inflammation [336]. It was the first FXR agonist to reach the clinical stage [337]. Investigations have demonstrated that obeticholic modified fat metabolism and improve insulin sensitivity as a result of the role FXR signaling plays in the control of lipid and glucose balance [19]. Similarly, TGR5 agonists enhance a myocardial reaction to stress in mice [209], hence they, as well as FXR agonists, can be innovative targets for the management of heart failure [17]. Furthermore, targeting BSH microbiota–host interplay could be a strategy for metabolic disorders and obesity since studies have shown that microbial BSH action may remarkably decrease weight gain as well as plasma cholesterol [338,339]. Statin drugs were demonstrated to affect the BA pool as well as decrease gut butyrate generation [340].

### 5.7. Targeting Other Metabolites/Enzymes

Significant advancement has been recorded in the comprehension of intestinal microbial metabolism. The exposition of more microbial products allows additional likely therapeutic targets for CMD [19]. The intestinal microbiota tryptophan decarboxylase was identified to be accountable for the generation of tryptamine [341]. Microbial dissimilatory sulfite reductases (DsrAB) are responsible for hydrogen sulfide production [342] while tryptophanases lead to indole generation [343]. The gut microbiota glycyl radical enzyme was also recognized to break the C-S bond of taurine to produce hydrogen sulfide [344]. The applicability of the mentioned microbial enzymes and the production of their inhibitors as innovative therapeutic targets require further studies. This is with the aim of improving the liberation of useful metabolites and attenuating the generation of harmful ones [19].

## 6. Challenges

The interplay between the host and the gut microbiota is usually dynamic and dictated by gastric motion, regional nutrient accessibility, pH, and oxygen pressure. Microbial ecology is usually different and distinctive along the intestinal tract. This creates problems in elucidating microbial make-up predicated on fecal analyses [345]. Furthermore, viruses, fungi, and archaea as well add to the non-host genetic information acquired during deep sequencing evaluation, aside from bacteria. This creates more complications in the microbial analyses and interpretations [23]. The differences in intestinal microbial ecology across various segments and individuals result in different reactions to probiotics and prebiotics [346]. This has therefore affected the predictability of the outcome of probiotic or prebiotic intake. Furthermore, the present choice of probiotics is based on an abundance evaluation of microbiota make-up where the focus is on the microorganism whose abundance is linked with favorable effects. However, the main microbial organism responsible for the key beneficial effect may be a low-abundance member that is not readily identified using the present sequencing analysis [21].

## 7. Future Direction: Microbiota in Precision Medicine

Due to the interindividual variation in the gut microbiome, the human microbiome has been associated with and has a promise in precision medicine [347]. The human microbiome is being seriously investigated as a therapeutic target through the deployment of the aforementioned approaches [80]. TMAO levels can be employed clinically to determine individuals who will benefit from CMD treatment [9]. The human microbiome is very dynamic and differences within and between individuals’ microbiota may influence drug efficacy and adverse effects. This is achieved indirectly through microbial–host immune interaction or directly through the biotransformation of drugs. Therefore, there are several upcoming approaches for the precise modulation of complex microbial ecology to enhance CMD treatment outcomes [80]. Hence, we expect a positive move regarding a comprehensive perspective of precision medicine that includes human as well as microbial genomes and their combined metabolites. Large clinical trials will be needed in the future to be able to translate the findings on targeting gut microbiota for cardiometabolic therapy into clinical practice.

## Data Availability

Not applicable.

## References

[1] Benjamin E.J., Virani S.S., Callaway C.W., Chamberlain A.M., Chang A.R., Cheng S., Chiuve S.E., Cushman M., Delling F.N., Deo R. (2018). Heart Disease and Stroke Statistics—2018 Update: A Report from the American Heart Association. Circulation.

[2] Guo F., Moellering D.R., Garvey W.T. (2013). The progression of cardiometabolic disease: Validation of a new cardiometabolic disease staging system applicable to obesity. Obesity.

[3] Benjamin E.J., Blaha M.J., Chiuve S.E., Cushman M., Das S.R., Deo R., de Ferranti S.D., Floyd J., Fornage M., Gillespie C. (2016). Heart disease and stroke statistics-2016 update a report from the American Heart Association. Circulation.

[4] Virani S.S., Alonso A., Benjamin E.J., Bittencourt M.S., Callaway C.W., Carson A.P., Chamberlain A.M., Chang A.R., Cheng S., Delling F.N. (2020). Heart Disease and Stroke Statistics—2020 Update: A Report from the American Heart Association. Circulation.

[5] Naghavi M., Wang H., Lozano R., Davis A., Liang X., Zhou M., Vollset S.E., Abbasoglu Ozgoren A., Abdalla S., Abd-Allah F. (2015). Global, regional, and national age-sex specific all-cause and cause-specific mortality for 240 causes of death, 1990–2013: A systematic analysis for the Global Burden of Disease Study. Lancet.

[6] Koren O., Spor A., Felin J., Fåk F., Stombaugh J., Tremaroli V., Behre C.J., Knight R., Fagerberg B., Ley R.E. (2011). Human oral, gut, and plaque microbiota in patients with atherosclerosis. Proc. Natl. Acad. Sci. USA.

[7] Karlsson F., Fåk F., Nookaew I., Tremaroli V., Fagerberg B., Petranovic D., Bäckhed F., Nielsen J. (2012). Symptomatic atherosclerosis is associated with an altered gut metagenome. Nat. Commun..

[8] Yamashiro K., Tanaka R., Urabe T., Ueno Y., Yamashiro Y., Nomoto K., Takahashi T., Tsuji H., Asahara T., Hattori N. (2017). Gut dysbiosis is associated with metabolism and systemic inflammation in patients with ischemic stroke. PLoS ONE.

[9] Xu H., Wang X., Feng W., Liu Q., Zhou S., Liu Q., Cai L. (2020). The gut microbiota and its interactions with cardiovascular disease. Microb. Biotechnol..

[10] Jin L., Shi X., Yang J., Zhao Y., Xue L., Xu L., Cai J. (2021). Gut microbes in cardiovascular diseases and their potential therapeutic applications. Protein Cell.

[11] Wilson Tang W.H., Hazen S.L. (2017). The Gut Microbiome and Its Role in Cardiovascular Diseases. Circulation.

[12] Tang W.W., Kitai T., Hazen S.L. (2017). Gut Microbiota in Cardiovascular Health and Disease. Circ. Res..

[13] Ríos-Covián D., Ruas-Madiedo P., Margolles A., Gueimonde M., De Los Reyes-Gavilán C.G., Salazar N. (2016). Intestinal Short Chain Fatty Acids and their Link with Diet and Human Health. Front. Microbiol..

[14] Tuteja S., Ferguson J.F. (2019). Gut Microbiome and Response to Cardiovascular Drugs. Circ. Genom. Precis. Med..

[15] Wikoff W.R., Anfora A.T., Liu J., Schultz P.G., Lesley S.A., Peters E.C., Siuzdak G. (2009). Metabolomics analysis reveals large effects of gut microflora on mammalian blood metabolites. Proc. Natl. Acad. Sci. USA.

[16] Brown J.M., Hazen S.L. (2015). The Gut Microbial Endocrine Organ: Bacterially Derived Signals Driving Cardiometabolic Diseases. Annu. Rev. Med..

[17] Jia Q., Li H., Zhou H., Zhang X., Zhang A., Xie Y., Li Y., Lv S., Zhang J. (2019). Role and Effective Therapeutic Target of Gut Microbiota in Heart Failure. Cardiovasc. Ther..

[18] Schuster H. (2004). Improving lipid management—To titrate, combine or switch. Int. J. Clin. Pract..

[19] Du Y., Li X., Su C., Wang L., Jiang J., Hong B. (2019). The human gut microbiome—A new and exciting avenue in cardiovascular drug discovery. Expert Opin. Drug Discov..

[20] Ejtahed H.-S., Soroush A.-R., Angoorani P., Larijani B., Hasani-Ranjbar S. (2016). Gut Microbiota as a Target in the Pathogenesis of Metabolic Disorders: A New Approach to Novel Therapeutic Agents. Horm. Metab. Res..

[21] Witkowski M., Weeks T.L., Hazen S.L. (2020). Gut Microbiota and Cardiovascular Disease. Circ. Res..

[22] Grice E.A., Segre J.A. (2012). Published in final edited form as: The Human Microbiome: Our Second Genome. Annu. Rev. Genomics Hum. Genet..

[23] Tang W.H.W., Bäckhed F., Landmesser U., Hazen S.L. (2019). Intestinal Microbiota in Cardiovascular Health and Disease: JACC State-of-the-Art Review. J. Am. Coll Cardiol..

[24] Sender R., Fuchs S., Milo R. (2016). Revised Estimates for the Number of Human and Bacteria Cells in the Body. PLOS Biol..

[25] (2012). Human Microbiome Project Consortium. Structure, function and diversity of the healthy human microbiome The Human Microbiome Project Consortium*. Nature.

[26] Qin J., Li R., Raes J., Arumugam M., Burgdorf K.S., Manichanh C., Nielsen T., Pons N., Levenez F., Yamada T. (2010). A human gut microbial gene catalog established by metagenomic sequencing. Nature.

[27] Zabell A., Tang W.H.W. (2017). Targeting the Microbiome in Heart Failure. Curr. Treat. Options Cardiovasc. Med..

[28] Sekirov I., Russell S.L., Antunes L.C.M., Finlay B.B. (2010). Gut Microbiota in Health and Disease. Physiol. Rev..

[29] Ranjan R., Rani A., Metwally A., McGee H.S., Perkins D.L. (2017). Analysis of the microbiome: Advantages of whole genome shotgun versus 16S amplicon sequencing. Biochem. Biophys. Res. Commun..

[30] Chen X., Li H.-Y., Hu X.-M., Zhang Y., Zhang S.-Y. (2019). Current understanding of gut microbiota alterations and related therapeutic intervention strategies in heart failure. Chin. Med J..

[31] Kitai Takeshi T.W.H.W. (2019). Gut Microbiota in Cardiovascular Disease and Heart Failure. Clin Sci..

[32] Nicholson J.K., Holmes E., Wilson I.D. (2005). Gut microorganisms, mammalian metabolism and personalized health care. Nat. Rev. Genet..

[33] Carding S., Verbeke K., Vipond D.T., Corfe B.M., Owen L.J. (2015). Dysbiosis of the gut microbiota in disease. Microb. Ecol. Health Dis..

[34] Lau K., Srivatsav V., Rizwan A., Nashed A., Liu R., Shen R., Akhtar M. (2017). Bridging the Gap between Gut Microbial Dysbiosis and Cardiovascular Diseases. Nutrients.

[35] Tremaroli V., Bäckhed F. (2012). Functional interactions between the gut microbiota and host metabolism. Nature.

[36] Wong J.M.W., de Souza R., Kendall C.W.C., Emam A., Jenkins D.J.A. (2006). Colonic Health: Fermentation and Short Chain Fatty Acids. J. Clin. Gastroenterol..

[37] den Besten G., Lange K., Havinga R., van Dijk T.H., Gerding A., van Eunen K., Müller M., Groen A.K., Hooiveld G.J., Bakker B.M. (2013). Gut-derived short-chain fatty acids are vividly assimilated into host carbohydrates and lipids. Am. J. Physiol.-Gastrointest. Liver Physiol..

[38] Coppola S., Avagliano C., Calignano A., Canani R.B. (2021). The Protective Role of Butyrate against Obesity and Obesity-Related Diseases. Molecules.

[39] Louis P., Flint H.J. (2017). Formation of propionate and butyrate by the human colonic microbiota. Environ. Microbiol..

[40] Oiso H., Furukawa N., Suefuji M., Shimoda S., Ito A., Furumai R., Nakagawa J., Yoshida M., Nishino N., Araki E. (2011). The role of class I histone deacetylase (HDAC) on gluconeogenesis in liver. Biochem. Biophys. Res. Commun..

[41] Khan S., Jena G. (2016). Sodium butyrate reduces insulin-resistance, fat accumulation and dyslipidemia in type-2 diabetic rat: A comparative study with metformin. Chem. Interactions.

[42] Christiansen C.B., Gabe M.B.N., Svendsen B., Dragsted L.O., Rosenkilde M.M., Holst J.J. (2018). The impact of short-chain fatty acids on GLP-1 and PYY secretion from the isolated perfused rat colon. Am. J. Physiol. Liver Physiol..

[43] Yadav H., Lee J.-H., Lloyd J., Walter P., Rane S.G. (2013). Beneficial Metabolic Effects of a Probiotic via Butyrate-induced GLP-1 Hormone Secretion. J. Biol. Chem..

[44] McBrayer D.N., Tal-Gan Y. (2017). Recent Advances in GLP-1 Receptor Agonists for Use in Diabetes Mellitus. Drug Dev. Res..

[45] Hong J., Jia Y., Pan S., Jia L., Li H., Han Z., Cai D., Zhao R. (2016). Butyrate alleviates high fat diet-induced obesity through activation of adiponectin-mediated pathway and stimulation of mitochondrial function in the skeletal muscle of mice. Oncotarget.

[46] Den Besten G., Bleeker A., Gerding A., van Eunen K., Havinga R., van Dijk T.H., Oosterveer M.H., Jonker J.W., Groen A.K., Reijngoud D.J. (2015). Short-Chain Fatty Acids Protect Against High-Fat Diet-Induced Obesity via a PPARγ-Dependent Switch from Lipogenesis to Fat Oxidation. Diabetes.

[47] Gao Z., Yin J., Zhang J., Ward R.E., Martin R.J., Lefevre M., Cefalu W.T., Ye J. (2009). Butyrate Improves Insulin Sensitivity and Increases Energy Expenditure in Mice. Diabetes.

[48] Li Z., Yi C.-X., Katiraei S., Kooijman S., Zhou E., Chung C.K., Gao Y., van den Heuvel J.K., Meijer O.C., Berbée J.F.P. (2018). Butyrate reduces appetite and activates brown adipose tissue via the gut-brain neural circuit. Gut.

[49] Tolhurst G., Heffron H., Lam Y.S., Parker H.E., Habib A.M., Diakogiannaki E., Cameron J., Grosse J., Reimann F., Gribble F.M. (2012). Short-Chain Fatty Acids Stimulate Glucagon-Like Peptide-1 Secretion via the G-Protein-Coupled Receptor FFAR. Diabetes.

[50] Lin H.V., Frassetto A., Kowalik E.J., Nawrocki A.R., Lu M.M., Kosinski J.R., Hubert J.A., Szeto D., Yao X., Forrest G. (2012). Butyrate and Propionate Protect against Diet-Induced Obesity and Regulate Gut Hormones via Free Fatty Acid Receptor 3-Independent Mechanisms. PLoS ONE.

[51] Kondo T., Kishi M., Fushimi T., Kaga T. (2009). Acetic Acid Upregulates the Expression of Genes for Fatty Acid Oxidation Enzymes in Liver To Suppress Body Fat Accumulation. J. Agric. Food Chem..

[52] Yamashita H., Fujisawa K., Ito E., Idei S., Kawaguchi N., Kimoto M., Hiemori M., Tsuji H. (2007). Improvement of Obesity and Glucose Tolerance by Acetate in Type 2 Diabetic Otsuka Long-Evans Tokushima Fatty (OLETF) Rats. Biosci. Biotechnol. Biochem..

[53] Yamashita H., Maruta H., Jozuka M., Kimura R., Iwabuchi H., Yamato M., Saito T., Fujisawa K., Takahashi Y., Kimoto M. (2009). Effects of Acetate on Lipid Metabolism in Muscles and Adipose Tissues of Type 2 Diabetic Otsuka Long-Evans Tokushima Fatty (OLETF) Rats. Biosci. Biotechnol. Biochem..

[54] Sahuri-Arisoylu M., Brody L.P., Parkinson J.R., Parkes H., Navaratnam N., Miller A.D., Thomas E.L., Frost G., Bell J.D. (2016). Reprogramming of hepatic fat accumulation and ‘browning’ of adipose tissue by the short-chain fatty acid acetate. Int. J. Obes..

[55] Cohen P., Levy J.D., Zhang Y., Frontini A., Kolodin D.P., Svensson K.J., Lo J.C., Zeng X., Ye L., Khandekar M.J. (2014). Ablation of PRDM16 and Beige Adipose Causes Metabolic Dysfunction and a Subcutaneous to Visceral Fat Switch. Cell.

[56] Harms M.J., Ishibashi J., Wang W., Lim H.-W., Goyama S., Sato T., Kurokawa M., Won K.-J., Seale P. (2014). Prdm16 Is Required for the Maintenance of Brown Adipocyte Identity and Function in Adult Mice. Cell Metab..

[57] De Vadder F., Kovatcheva-Datchary P., Goncalves D., Vinera J., Zitoun C., Duchampt A., Bäckhed F., Mithieux G. (2014). Microbiota-generated metabolites promote metabolic benefits via gut-brain neural circuits. Cell.

[58] Mithieux G., Misery P., Magnan C., Pillot B., Gautier-Stein A., Bernard C., Rajas F., Zitoun C. (2005). Portal sensing of intestinal gluconeogenesis is a mechanistic link in the diminution of food intake induced by diet protein. Cell Metab..

[59] Simpson H.L., Campbell B.J. (2015). Review article: Dietary fibre-microbiota interactions. Aliment. Pharmacol. Ther..

[60] Singh R.K., Chang H.-W., Yan D., Lee K.M., Ucmak D., Wong K., Abrouk M., Farahnik B., Nakamura M., Zhu T.H. (2017). Influence of diet on the gut microbiome and implications for human health. J. Transl. Med..

[61] Bäckhed F., Ley R.E., Sonnenburg J.L., Peterson D.A., Gordon J.I. (2005). Host-Bacterial Mutualism in the Human Intestine. Science.

[62] Bäckhed F., Ding H., Wang T., Hooper L.V., Koh G.Y., Nagy A., Semenkovich C.F., Gordon J.I. (2004). The Gut Microbiota as an Environmental Factor That Regulates Fat Storage. Proc. Natl. Acad. Sci. USA.

[63] Mazmanian S.K., Liu C.H., Tzianabos A.O., Kasper D.L. (2005). An Immunomodulatory Molecule of Symbiotic Bacteria Directs Maturation of the Host Immune System. Cell.

[64] Li M., Wang B., Zhang M., Rantalainen M., Wang S., Zhou H., Zhang Y., Shen J., Pang X., Zhang M. (2008). Symbiotic gut microbes modulate human metabolic phenotypes. Proc. Natl. Acad. Sci. USA.

[65] Quigley E.M.M., Eamonn D., Quigley M.M. (2013). Gut Bacteria in Health and Disease. Gastroenterol. Hepatol..

[66] Kamada N., Seo S.-U., Chen G.Y., Núñez G. (2013). Role of the gut microbiota in immunity and inflammatory disease. Nat. Rev. Immunol..

[67] (2008). News & Highlights. Mucosal Immunol..

[68] Cerf-Bensussan N., Gaboriau-Routhiau V. (2010). The immune system and the gut microbiota: Friends or foes?. Nat. Rev. Immunol..

[69] Kitai T., Kirsop J., Tang W.H.W. (2016). Exploring the Microbiome in Heart Failure. Curr. Heart Fail. Rep..

[70] Chiang J.Y.L. (2013). Bile Acid Metabolism and Signaling. Compr. Physiol..

[71] Lyte M. (2011). Probiotics function mechanistically as delivery vehicles for neuroactive compounds: Microbial endocrinology in the design and use of probiotics. BioEssays.

[72] Mahmoodpoor F., Rahbar Saadat Y., Barzegari A., Ardalan M., Zununi Vahed S. (2017). The impact of gut microbiota on kidney function and pathogenesis. Biomed. Pharmacother..

[73] Sandek A., Bauditz J., Swidsinski A., Buhner S., Weber-Eibel J., von Haehling S., Schroedl W., Karhausen T., Doehner W., Rauchhaus M. (2007). Altered Intestinal Function in Patients with Chronic Heart Failure. J. Am. Coll. Cardiol..

[74] Tang W.W., Hazen S.L. (2014). The contributory role of gut microbiota in cardiovascular disease. J. Clin. Investig..

[75] Sobko T., Huang L., Midtvedt T., Norin E., Gustafsson L.E., Norman M., Jansson E., Lundberg J.O. (2006). Generation of NO by probiotic bacteria in the gastrointestinal tract. Free Radic. Biol. Med..

[76] Allayee H., Hazen S.L. (2015). Contribution of Gut Bacteria to Lipid Levels: Another Metabolic Role for Microbes?. Circ. Res..

[77] Afsar B., Vaziri N.D., Aslan G., Tarim K., Kanbay M. (2016). Gut hormones and gut microbiota: Implications for kidney function and hypertension. J. Am. Soc. Hypertens..

[78] Nutting C.W., Islam S., Daugirdas J.T. (1991). Vasorelaxant effects of short chain fatty acid salts in rat caudal artery. Am. J. Physiol. Circ. Physiol..

[79] de Andrade J.A.A., Gayer C.R.M., Nogueira N.P.D.A., Paes M.C., Bastos V.L.F.C., Neto J.D.C.B., Alves S.C., Coelho R.M., da Cunha M.G.A.T., Gomes R.N. (2014). The effect of thiamine deficiency on inflammation, oxidative stress and cellular migration in an experimental model of sepsis. J. Inflamm..

[80] Kazemian N., Mahmoudi M., Halperin F., Wu J.C., Pakpour S. (2020). Gut microbiota and cardiovascular disease: Opportunities and challenges. Microbiome.

[81] Tremaroli V., Karlsson F., Werling M., Ståhlman M., Kovatcheva-Datchary P., Olbers T., Fändriks L., le Roux C.W., Nielsen J., Bäckhed F. (2015). Roux-en-Y Gastric Bypass and Vertical Banded Gastroplasty Induce Long-Term Changes on the Human Gut Microbiome Contributing to Fat Mass Regulation. Cell Metab..

[82] Serino M., Blasco-Baque V., Nicolas S., Burcelin R. (2014). Far from the Eyes, Close to the Heart: Dysbiosis of Gut Microbiota and Cardiovascular Consequences. Curr. Cardiol. Rep..

[83] Hawrelak J.A., Myers S.P. (2004). The causes of intestinal dysbiosis: A review. Altern. Med. Rev..

[84] Garrett W.S. (2015). Cancer and the microbiota. Science.

[85] Zhao W., Caro F., Robins W., Mekalanos J.J. (2018). Antagonism toward the intestinal microbiota and its effect on *Vibrio cholerae* virulence. Science.

[86] Chu H., Khosravi A., Kusumawardhani I.P., Kwon A.H.K., Vasconcelos A.C., Cunha L.D., Mayer A.E., Shen Y., Wu W.-L., Kambal A. (2016). Gene-microbiota interactions contribute to the pathogenesis of inflammatory bowel disease. Science.

[87] Zhang C., Yin A., Li H., Wang R., Wu G., Shen J., Zhang M., Wang L., Hou Y., Ouyang H. (2015). Dietary Modulation of Gut Microbiota Contributes to Alleviation of Both Genetic and Simple Obesity in Children. eBioMedicine.

[88] Zhu L., Baker S.S., Gill C., Liu W., Alkhouri R., Baker R.D., Gill S.R. (2013). Characterization of gut microbiomes in nonalcoholic steatohepatitis (NASH) patients: A connection between endogenous alcohol and NASH. Hepatology.

[89] Zhang X., Zhang D., Jia H., Feng Q., Wang D., Liang D., Wu X., Li J., Tang L., Li Y. (2015). The oral and gut microbiomes are perturbed in rheumatoid arthritis and partly normalized after treatment. Nat. Med..

[90] Kang D.-W., Adams J.B., Gregory A.C., Borody T., Chittick L., Fasano A., Khoruts A., Geis E., Maldonado J., McDonough-Means S. (2017). Microbiota Transfer Therapy alters gut ecosystem and improves gastrointestinal and autism symptoms: An open-label study. Microbiome.

[91] Kim S., Goel R., Kumar A., Qi Y., Lobaton G., Hosaka K., Mohammed M., Handberg E., Richards E.M., Pepine C.J. (2018). Imbalance of gut microbiome and intestinal epithelial barrier dysfunction in patients with high blood pressure. Clin. Sci..

[92] Karbach S.H., Schönfelder T., Brandão I., Wilms E., Hörmann N., Jäckel S., Schüler R., Finger S., Knorr M., Lagrange J. (2016). Gut Microbiota Promote Angiotensin II–Induced Arterial Hypertension and Vascular Dysfunction. J. Am. Heart Assoc..

[93] Yin J., Liao S.X., He Y., Wang S., Xia G.H., Liu F.T., Zhu J.J., You C., Chen Q., Zhou L. (2015). Dysbiosis of Gut Microbiota With Reduced Trimethylamine-N-Oxide Level in Patients With Large-Artery Atherosclerotic Stroke or Transient Ischemic Attack. J. Am. Hear. Assoc..

[94] Li J., Zhao F., Wang Y., Chen J., Tao J., Tian G., Wu S., Liu W., Cui Q., Geng B. (2017). Gut microbiota dysbiosis contributes to the development of hypertension. Microbiome.

[95] Wang Z., Roberts A.B., Buffa J.A., Levison B.S., Zhu W., Org E., Gu X., Huang Y., Zamanian-Daryoush M., Culley M.K. (2015). Non-lethal Inhibition of Gut Microbial Trimethylamine Production for the Treatment of Atherosclerosis. Cell.

[96] Zhu W., Gregory J.C., Org E., Buffa J.A., Gupta N., Wang Z., Li L., Fu X., Wu Y., Mehrabian M. (2016). Gut Microbial Metabolite TMAO Enhances Platelet Hyperreactivity and Thrombosis Risk. Cell.

[97] Cui X., Ye L., Li J., Jin L., Wang W., Li S., Bao M., Wu S., Li L., Geng B. (2018). Metagenomic and metabolomic analyses unveil dysbiosis of gut microbiota in chronic heart failure patients. Sci. Rep..

[98] Emoto T., Yamashita T., Sasaki N., Hirota Y., Hayashi T., So A., Kasahara K., Yodoi K., Matsumoto T., Mizoguchi T. (2016). Analysis of Gut Microbiota in Coronary Artery Disease Patients: A Possible Link between Gut Microbiota and Coronary Artery Disease. J. Atheroscler. Thromb..

[99] Wong J.M.W. (2014). Gut microbiota and cardiometabolic outcomes: Influence of dietary patterns and their associated components. Am. J. Clin. Nutr..

[100] Mariat D., Firmesse O., Levenez F., Guimaraes V.D., Sokol H., Dore J., Corthier G., Furet J.-P. (2009). The Firmicutes/Bacteroidetes ratio of the human microbiota changes with age. BMC Microbiol..

[101] Sanz Y., Moya-Pérez A. (2014). Microbiota, inflammation and obesity. Adv. Exp. Med. Biol..

[102] Gózd-Barszczewska A., Kozioł-Montewka M., Barszczewski P., Młodzińska A., Humińska K. (2017). Gut microbiome as a biomarker of cardiometabolic disorders. Ann. Agric. Environ. Med..

[103] Yan Q., Gu Y., Li X., Yang W., Jia L., Chen C., Han X., Huang Y., Zhao L., Li P. (2017). Alterations of the Gut Microbiome in Hypertension. Front. Cell. Infect. Microbiol..

[104] Wilck N., Matus M.G., Kearney S.M., Olesen S.W., Forslund K., Bartolomaeus H., Haase S., Mähler A., Balogh A., Markó L. (2017). Salt-responsive gut commensal modulates TH17 axis and disease. Nature.

[105] Pasini E., Aquilani R., Testa C., Baiardi P., Angioletti S., Boschi F., Verri M., Dioguardi F.S. (2016). Pathogenic Gut Flora in Patients With Chronic Heart Failure. JACC Heart Fail..

[106] Qin J., Li Y., Cai Z., Li S., Zhu J., Zhang F., Liang S., Zhang W., Guan Y., Shen D. (2012). A metagenome-wide association study of gut microbiota in type 2 diabetes. Nature.

[107] Jie Z., Xia H., Zhong S.-L., Feng Q., Li S., Liang S., Zhong H., Liu Z., Gao Y., Zhao H. (2017). The gut microbiome in atherosclerotic cardiovascular disease. Nat. Commun..

[108] Emoto T., Yamashita T., Kobayashi T., Sasaki N., Hirota Y., Hayashi T., So A., Kasahara K., Yodoi K., Matsumoto T. (2017). Characterization of gut microbiota profiles in coronary artery disease patients using data mining analysis of terminal restriction fragment length polymorphism: Gut microbiota could be a diagnostic marker of coronary artery disease. Heart Vessel..

[109] Ott S.J., El Mokhtari N.E., Musfeldt M., Hellmig S., Freitag S., Rehman A., Kuhbacher T., Nikolaus S., Namsolleck P., Blaut M. (2006). Detection of Diverse Bacterial Signatures in Atherosclerotic Lesions of Patients with Coronary Heart Disease. Circulation.

[110] Shin N.R., Lee J.C., Lee H.Y., Kim M.S., Whon T.W., Lee M.S., Bae J.W. (2014). An increase in the *Akkermansia* spp. population induced by metformin treatment improves glucose homeostasis in diet-induced obese mice. Gut.

[111] Everard A., Belzer C., Geurts L., Ouwerkerk J.P., Druart C., Bindels L.B., Guiot Y., Derrien M., Muccioli G.G., Delzenne N.M. (2013). Cross-talk between *Akkermansia muciniphila* and intestinal epithelium controls diet-induced obesity. Proc. Natl. Acad. Sci. USA.

[112] Derrien M., Vaughan E.E., Plugge C.M., De Vos W.M. (2004). Akkermansia muciniphila gen. nov., sp. nov., a human intestinal mucin-degrading bacterium. Int. J. Syst. Evol. Microbiol..

[113] Lau K., Benitez P., Ardissone A., Wilson T.D., Collins E.L., Lorca G., Li N., Sankar D., Wasserfall C., Neu J. (2011). Inhibition of Type 1 Diabetes Correlated to a Lactobacillus johnsonii N6.2-Mediated Th17 Bias. J. Immunol..

[114] Larsson E., Tremaroli V., Lee Y.S., Koren O., Nookaew I., Fricker A., Nielsen J., Ley R., Bäckhed F. (2012). Analysis of gut microbial regulation of host gene expression along the length of the gut and regulation of gut microbial ecology through MyD. Gut.

[115] Mitra S., Drautz-Moses D.I., Alhede M., Maw M.T., Liu Y., Purbojati R.W., Yap Z.H., Kushwaha K.K., Gheorghe A.G., Bjarnsholt T. (2015). In silico analyses of metagenomes from human atherosclerotic plaque samples. Microbiome.

[116] Jonsson A.L., Bäckhed F. (2017). Role of gut microbiota in atherosclerosis. Nat. Rev. Cardiol..

[117] Lawler P.R., Bhatt D.L., Godoy L.C., Lüscher T.F., O Bonow R., Verma S., Ridker P.M. (2021). Targeting cardiovascular inflammation: Next steps in clinical translation. Eur. Heart J..

[118] Geovanini G.R., Libby P. (2018). Atherosclerosis and inflammation: Overview and updates. Clin. Sci..

[119] Libby P. (2012). Inflammation in Atherosclerosis. Arter. Thromb. Vasc. Biol..

[120] Ridker P.M., Rifai N., Clearfield M., Downs J.R., Weis S.E., Miles J.S., Gotto A.M. (2001). Measurement of C-Reactive Protein for the Targeting of Statin Therapy in the Primary Prevention of Acute Coronary Events. N. Engl. J. Med..

[121] Ridker P.M., Everett B.M., Thuren T., MacFadyen J.G., Chang W.H., Ballantyne C., Fonseca F., Nicolau J., Koenig W., Anker S.D. (2017). Antiinflammatory Therapy with Canakinumab for Atherosclerotic Disease. N. Engl. J. Med..

[122] Zheng Y., Valdez P.A., Danilenko D.M., Hu Y., Sa S.M., Gong Q., Abbas A.R., Modrusan Z., Ghilardi N., De Sauvage F.J. (2008). Interleukin-22 mediates early host defense against attaching and effacing bacterial pathogens. Nat. Med..

[123] Sugimoto K., Ogawa A., Mizoguchi E., Shimomura Y., Andoh A., Bhan A.K., Blumberg R.S., Xavier R.J., Mizoguchi A. (2008). IL-22 ameliorates intestinal inflammation in a mouse model of ulcerative colitis. J. Clin. Investig..

[124] Wang X., Ota N., Manzanillo P., Kates L., Zavala-Solorio J., Eidenschenk C., Zhang J., Lesch J., Lee W.P., Ross J. (2014). Interleukin-22 alleviates metabolic disorders and restores mucosal immunity in diabetes. Nature.

[125] Wang Z., Klipfell E., Bennett B.J., Koeth R., Levison B.S., DuGar B., Feldstein A.E., Britt E.B., Fu X., Chung Y.-M. (2011). Gut Flora Metabolism of Phosphatidylcholine Promotes Cardiovascular Disease. Nature.

[126] Furusawa Y., Obata Y., Fukuda S., Endo T.A., Nakato G., Takahashi D., Nakanishi Y., Uetake C., Kato K., Kato T. (2013). Commensal microbe-derived butyrate induces the differentiation of colonic regulatory T cells. Nature.

[127] Seldin M.M., Meng Y., Qi H., Zhu W., Wang Z., Hazen S.L., Lusis A.J., Shih D.M. (2016). Trimethylamine N-Oxide Promotes Vascular Inflammation Through Signaling of Mitogen-Activated Protein Kinase and Nuclear Factor-κB. J. Am. Heart Assoc..

[128] Koeth R.A., Wang Z., Levison B.S., Buffa J.A., Org E., Sheehy B.T., Britt E.B., Fu X., Wu Y., Li L. (2013). Intestinal microbiota metabolism of l-carnitine, a nutrient in red meat, promotes atherosclerosis. Nat. Med..

[129] Blacher E., Levy M., Tatirovsky E., Elinav E. (2017). Microbiome-Modulated Metabolites at the Interface of Host Immunity. J. Immunol..

[130] Royall D., Wolever T.M., Jeejeebhoy K.N. (1990). Clinical significance of colonic fermentation. Am. J. Gastroenterol..

[131] Corrêa-Oliveira R., Fachi J.L., Vieira A., Sato F.T., Vinolo M.A.R. (2016). Regulation of immune cell function by short-chain fatty acids. Clin. Transl. Immunol..

[132] Yang T., Santisteban M.M., Rodriguez V., Li E., Ahmari N., Carvajal J.M., Zadeh M., Gong M., Qi Y., Zubcevic J. (2015). Gut Dysbiosis Is Linked to Hypertension. Hypertension.

[133] Mell B., Jala V.R., Mathew A.V., Byun J., Waghulde H., Zhang Y., Haribabu B., Vijay-Kumar M., Pennathur S., Joe B. (2015). Evidence for a link between gut microbiota and hypertension in the Dahl rat. Physiol. Genomics.

[134] Pluznick J.L., Protzko R.J., Gevorgyan H., Peterlin Z., Sipos A., Han J., Brunet I., Wan L.X., Rey F., Wang T. (2013). Olfactory receptor responding to gut microbiota derived signals plays a role in renin secretion and blood pressure regulation. Proc. Natl. Acad. Sci. USA.

[135] Wang L., Zhu Q., Lu A., Liu X., Zhang L., Xu C., Liu X., Li H., Yang T. (2017). Sodium butyrate suppresses angiotensin II-induced hypertension by inhibition of renal (pro)renin receptor and intrarenal renin–angiotensin system. J. Hypertens..

[136] Krishnan S., Alden N., Lee K. (2015). Pathways and functions of gut microbiota metabolism impacting host physiology. Curr. Opin. Biotechnol..

[137] De Preter V., Coopmans T., Rutgeerts P., Verbeke K. (2006). Influence of long-term administration of lactulose and Saccharomyces boulardii on the colonic generation of phenolic compounds in healthy human subjects. J. Am. Coll Nutr..

[138] Berni Canani R., Di Costanzo M., Leone L. (2012). The epigenetic effects of butyrate: Potential therapeutic implications for clinical practice. Clin. Epigenetics.

[139] Henagan T.M., Stefanska B., Fang Z., Navard A.M., Ye J., Lenard N.R., Devarshi P. (2015). Sodium butyrate epigenetically modulates high-fat diet-induced skeletal muscle mitochondrial adaptation, obesity and insulin resistance through nucleosome positioning. J. Cereb. Blood Flow Metab..

[140] Vinolo M.A., Rodrigues H.G., Nachbar R.T., Curi R. (2011). Regulation of Inflammation by Short Chain Fatty Acids. Nutrients.

[141] Karlsson F.H., Tremaroli V., Nookaew I., Bergström G., Behre C.J., Fagerberg B., Nielsen J., Bäckhed F. (2013). Gut metagenome in European women with normal, impaired and diabetic glucose control. Nature.

[142] Larsen N., Vogensen F.K., Van Den Berg F.W.J., Nielsen D.S., Andreasen A.S., Pedersen B.K., Al-Soud W.A., Sørensen S.J., Hansen L.H., Jakobsen M. (2010). Gut Microbiota in Human Adults with Type 2 Diabetes Differs from Non-Diabetic Adults. PLoS ONE.

[143] Kasselman L.J., Vernice N.A., DeLeon J., Reiss A.B. (2018). The gut microbiome and elevated cardiovascular risk in obesity and autoimmunity. Atherosclerosis.

[144] Marques F.Z., Nelson E., Chu P.-Y., Horlock D., Fiedler A., Ziemann M., Tan J.K., Kuruppu S., Rajapakse N.W., El-Osta A. (2017). High-Fiber Diet and Acetate Supplementation Change the Gut Microbiota and Prevent the Development of Hypertension and Heart Failure in Hypertensive Mice. Circulation.

[145] Toral M., Romero M., Rodríguez-Nogales A., Jimenez R., Robles-Vera I., Algieri F., Chueca-Porcuna N., Sánchez M., de la Visitación N., Olivares M. (2018). Lactobacillus fermentum Improves Tacrolimus-Induced Hypertension by Restoring Vascular Redox State and Improving eNOS Coupling. Mol. Nutr. Food Res..

[146] Kasahara K., Krautkramer K.A., Org E., Romano K.A., Kerby R.L., Vivas E.I., Mehrabian M., Denu J.M., Bäckhed F., Lusis A.J. (2018). Interactions between Roseburia intestinalis and diet modulate atherogenesis in a murine model. Nat. Microbiol..

[147] Brown A.J., Goldsworthy S.M., Barnes A.A., Eilert M.M., Tcheang L., Daniels D., Muir A.I., Wigglesworth M.J., Kinghorn I., Fraser N.J. (2003). The Orphan G Protein-coupled Receptors GPR41 and GPR43 Are Activated by Propionate and Other Short Chain Carboxylic Acids. J. Biol. Chem..

[148] Pluznick J.L. (2013). Renal and cardiovascular sensory receptors and blood pressure regulation. Am. J. Physiol. Physiol..

[149] Natarajan N., Hori D., Flavahan S., Steppan J., Flavahan N.A., Berkowitz D.E., Pluznick J.L. (2016). Microbial short chain fatty acid metabolites lower blood pressure via endothelial G protein-coupled receptor. Physiol. Genom..

[150] Marques F.Z., Mackay C.R., Kaye D.M. (2018). Beyond gut feelings: How the gut microbiota regulates blood pressure. Nat. Rev. Cardiol..

[151] Pluznick J.L. (2014). A novel SCFA receptor, the microbiota, and blood pressure regulation. Gut Microbes.

[152] Chang Y., Chen Y., Zhou Q., Wang C., Chen L., Di W., Zhang Y. (2020). Short-chain fatty acids accompanying changes in the gut microbiome contribute to the development of hypertension in patients with preeclampsia. Clin. Sci..

[153] Pluznick J.L. (2017). Microbial Short-Chain Fatty Acids and Blood Pressure Regulation. Curr. Hypertens. Rep..

[154] Frost G., Sleeth M.L., Sahuri-Arisoylu M., Lizarbe B., Cerdan S., Brody L., Anastasovska J., Ghourab S., Hankir M., Zhang S. (2014). The short-chain fatty acid acetate reduces appetite via a central homeostatic mechanism. Nat. Commun..

[155] Byrne C.S., Chambers E.S., Morrison D.J., Frost G. (2015). The role of short chain fatty acids in appetite regulation and energy homeostasis. Int. J. Obes..

[156] Kelly C.J., Zheng L., Campbell E.L., Saeedi B., Scholz C.C., Bayless A.J., Wilson K.E., Glover L.E., Kominsky D.J., Magnuson A. (2015). Crosstalk between Microbiota-Derived Short-Chain Fatty Acids and Intestinal Epithelial HIF Augments Tissue Barrier Function. Cell Host Microbe.

[157] Rath S., Heidrich B., Pieper D.H., Vital M. (2017). Uncovering the trimethylamine-producing bacteria of the human gut microbiota. Microbiome.

[158] Craciun S., Balskus E.P. (2012). Microbial conversion of choline to trimethylamine requires a glycyl radical enzyme. Proc. Natl. Acad. Sci. USA.

[159] Bennett B.J., de Aguiar Vallim T.Q., Wang Z., Shih D.M., Meng Y., Gregory J., Allayee H., Lee R., Graham M., Crooke R. (2013). Trimethylamine-N-Oxide, a Metabolite Associated with Atherosclerosis, Exhibits Complex Genetic and Dietary Regulation. Cell Metab..

[160] Zhu Y., Jameson E., Crosatti M., Schäfer H., Rajakumar K., Bugg T.D.H., Chen Y. (2014). Carnitine metabolism to trimethylamine by an unusual Rieske-type oxygenase from human microbiota. Proc. Natl. Acad. Sci. USA.

[161] Andreesen J.R. (1994). Glycine metabolism in anaerobes. Antonie Van Leeuwenhoek.

[162] Pascal M.-C., Burini J.-F., Chippaux M. (1984). Regulation of the trimethylamine N-oxide (TMAO) reductase in Escherichia coli: Analysis of tor::Mud1 operon fusion. Mol. Gen. Genet..

[163] Koeth R.A., Levison B.S., Culley M.K., Buffa J.A., Wang Z., Gregory J.C., Org E., Wu Y., Li L., Smith J.D. (2014). γ-Butyrobetaine Is a Proatherogenic Intermediate in Gut Microbial Metabolism of L-Carnitine to TMAO. Cell Metab..

[164] Ayesh R., Smith R.L. (1990). Genetic polymorphism of trimethylamine N-oxidation. Pharmacol. Ther..

[165] Fennema D., Phillips I.R., Shephard E.A. (2016). Trimethylamine and Trimethylamine N-Oxide, a Flavin-Containing Monooxygenase 3 (FMO3)-Mediated Host-Microbiome Metabolic Axis Implicated in Health and Disease. Drug Metab. Dispos..

[166] Warrier M., Shih D.M., Burrows A.C., Ferguson D., Gromovsky A.D., Brown A.L., Marshall S., McDaniel A., Schugar R.C., Wang Z. (2015). The TMAO-Generating Enzyme Flavin Monooxygenase 3 Is a Central Regulator of Cholesterol Balance. Cell Rep..

[167] Randrianarisoa E., Lehn-Stefan A., Wang X., Hoene M., Peter A., Heinzmann S.S., Zhao X., Königsrainer I., Königsrainer A., Balletshofer B. (2016). Relationship of Serum Trimethylamine N-Oxide (TMAO) Levels with early Atherosclerosis in Humans. Sci. Rep..

[168] Martin F.-P., Wang Y., Sprenger N., Yap I.K.S., Lundstedt T., Lek P., Rezzi S., Ramadan Z., Van Bladeren P., Fay L.B. (2008). Probiotic modulation of symbiotic gut microbial–host metabolic interactions in a humanized microbiome mouse model. Mol. Syst. Biol..

[169] Falony G., Vieira-Silva S., Raes J. (2015). Microbiology Meets Big Data: The Case of Gut Microbiota–Derived Trimethylamine. Annu. Rev. Microbiol..

[170] Wang Z., Bergeron N., Levison B.S., Li X.S., Chiu S., Jia X., Koeth R.A., Li L., Wu Y., Tang W.H.W. (2019). Impact of chronic dietary red meat, white meat, or non-meat protein on trimethylamine N-oxide metabolism and renal excretion in healthy men and women. Eur. Heart J..

[171] Bergeron N., Williams P.T., Lamendella R., Faghihnia N., Grube A., Li X., Wang Z., Knight R., Jansson J.K., Hazen S.L. (2016). Diets high in resistant starch increase plasma levels of trimethylamine-N-oxide, a gut microbiome metabolite associated with CVD risk. Br. J. Nutr..

[172] Brunt V.E., Gioscia-Ryan R.A., Casso A.G., VanDongen N.S., Ziemba B.P., Sapinsley Z.J., Richey J.J., Zigler M.C., Neilson A.P., Davy K.P. (2020). Trimethylamine-N-Oxide Promotes Age-Related Vascular Oxidative Stress and Endothelial Dysfunction in Mice and Healthy Humans. Hypertension.

[173] Tang W.H.W., Wang Z., Levison B.S., Koeth R.A., Britt E.B., Fu X., Wu Y., Hazen S.L. (2013). Intestinal Microbial Metabolism of Phosphatidylcholine and Cardiovascular Risk. N. Engl. J. Med..

[174] Tang W.W., Wang Z., Shrestha K., Borowski A.G., Wu Y., Troughton R.W., Klein A.L., Hazen S.L. (2015). Intestinal Microbiota-Dependent Phosphatidylcholine Metabolites, Diastolic Dysfunction, and Adverse Clinical Outcomes in Chronic Systolic Heart Failure. J. Card. Fail..

[175] Wang Z., Tang W.H.W., Buffa J.A., Fu X., Britt E.B., Koeth R.A., Levison B., Fan Y., Wu Y., Hazen S.L. (2014). Prognostic value of choline and betaine depends on intestinal microbiota-generated metabolite trimethylamine-N-oxide. Eur. Heart J..

[176] Senthong V., Wang Z., Fan Y., Wu Y., Hazen S.L., Tang W.H.W. (2016). Trimethylamine *N* -Oxide and Mortality Risk in Patients with Peripheral Artery Disease. J. Am. Heart Assoc..

[177] Senthong V., Wang Z., Li X.S., Fan Y., Wu Y., Wilson Tang W.H., Hazen S.L. (2016). Intestinal Microbiota-Generated Metabolite Trimethylamine-N-Oxide and 5-Year Mortality Risk in Stable Coronary Artery Disease: The Contributory Role of Intestinal Microbiota in a COURAGE-Like Patient Cohort. J. Am. Heart Assoc. Cardiovasc. Cerebrovasc. Dis..

[178] Li X.S., Obeid S., Klingenberg R., Gencer B., Mach F., Räber L., Windecker S., Rodondi N., Nanchen D., Muller O. (2017). Gut microbiota-dependent trimethylamine N-oxide in acute coronary syndromes: A prognostic marker for incident cardiovascular events beyond traditional risk factors. Eur. Heart J..

[179] Tang W.W., Wang Z., Fan Y., Levison B., Hazen J.E., Donahue L.M., Wu Y., Hazen S.L. (2014). Prognostic value of elevated levels of intestinal microbe-generated metabolite trimethylamine-N-oxide in patients with heart failure: Refining the gut hypothesis. J. Am. Coll. Cardiol..

[180] Romano K.A., Vivas E.I., Amador-Noguez D., Rey F.E. (2015). Intestinal Microbiota Composition Modulates Choline Bioavailability from Diet and Accumulation of the Proatherogenic Metabolite Trimethylamine-*N*-Oxide. mBio.

[181] Ge X., Zheng L., Zhuang R., Yu P., Xu Z., Liu G., Xi X., Zhou X., Fan H. (2020). The Gut Microbial Metabolite Trimethylamine N-Oxide and Hypertension Risk: A Systematic Review and Dose–Response Meta-analysis. Adv. Nutr. Int. Rev. J..

[182] Calderón-Pérez L., Gosalbes M.J., Yuste S., Valls R.M., Pedret A., Llauradó E., Jimenez-Hernandez N., Artacho A., Pla-Pagà L., Companys J. (2020). Gut metagenomic and short chain fatty acids signature in hypertension: A cross-sectional study. Sci. Rep..

[183] Nie J., Xie L., Zhao B.-X., Li Y., Qiu B., Zhu F., Li G.-F., He M., Wang Y., Wang B. (2018). Serum Trimethylamine N-Oxide Concentration Is Positively Associated with First Stroke in Hypertensive Patients. Stroke.

[184] Hauet T., Baumert H., Gibelin H., Hameury F., Goujon J.M., Carretier M., Eugene M. (2000). Noninvasive Monitoring of Citrate, Acetate, Lactate, and Renal Medullary Osmolyte Excretion in Urine as Biomarkers of Exposure to Ischemic Reperfusion Injury. Cryobiology.

[185] Griffin J.L., Wang X., Stanley E. (2015). Does Our Gut Microbiome Predict Cardiovascular Risk?. Circ. Cardiovasc. Genet..

[186] Ghazalpour A., Cespedes I., Bennett B.J., Allayee H. (2016). Expanding role of gut microbiota in lipid metabolism. Curr. Opin. Lipidol..

[187] Al-Obaide M.A.I., Singh R., Datta P., Rewers-Felkins K.A., Salguero M.V., Al-Obaidi I., Kottapalli K.R., Vasylyeva T.L. (2017). Gut Microbiota-Dependent Trimethylamine-N-oxide and Serum Biomarkers in Patients with T2DM and Advanced CKD. J. Clin. Med..

[188] Ma G., Pan B., Chen Y., Guo C., Zhao M., Zheng L., Chen B. (2017). Trimethylamine N-oxide in atherogenesis: Impairing endothelial self-repair capacity and enhancing monocyte adhesion. Biosci. Rep..

[189] Hansson G.K., Robertson A.K.L., Söderberg-Nauclér C. (2006). Inflammation and atherosclerosis. Annu. Rev. Pathol..

[190] Jones M.L., Martoni C.J., Ganopolsky J.G., Labbé A., Prakash S. (2014). The human microbiome and bile acid metabolism: Dysbiosis, dysmetabolism, disease and intervention. Expert Opin. Biol. Ther..

[191] Suzuki T., Heaney L.M., Bhandari S.S., Jones D.J.L., Ng L.L. (2016). Trimethylamine *N*-oxide and prognosis in acute heart failure. Heart.

[192] Trøseid M., Ueland T., Hov J., Svardal A., Gregersen I., Dahl C.P., Aakhus S., Gude E., Bjørndal B., Halvorsen B. (2015). Microbiota-dependent metabolite trimethylamine-N-oxide is associated with disease severity and survival of patients with chronic heart failure. J. Intern. Med..

[193] Li Z., Wu Z., Yan J., Liu H., Liu Q., Deng Y., Ou C., Chen M. (2019). Gut microbe-derived metabolite trimethylamine N-oxide induces cardiac hypertrophy and fibrosis. Lab. Investig..

[194] Jones B.V., Begley M., Hill C., Gahan C.G.M., Marchesi J.R. (2008). Functional and comparative metagenomic analysis of bile salt hydrolase activity in the human gut microbiome. Proc. Natl. Acad. Sci. USA.

[195] Jones M.L., Martoni C.J., Parent M., Prakash S. (2012). Cholesterol-lowering efficacy of a microencapsulated bile salt hydrolase-active *Lactobacillus reuteri* NCIMB 30242 yoghurt formulation in hypercholesterolaemic adults. Br. J. Nutr..

[196] Inagaki T., Choi M., Moschetta A., Peng L., Cummins C.L., McDonald J.G., Luo G., Jones S.A., Goodwin B., Richardson J.A. (2005). Fibroblast growth factor 15 functions as an enterohepatic signal to regulate bile acid homeostasis. Cell Metab..

[197] Pereira D.I.A., Gibson G.R. (2002). Effects of Consumption of Probiotics and Prebiotics on Serum Lipid Levels in Humans. Crit. Rev. Biochem. Mol. Biol..

[198] Haeusler R.A., Astiarraga B., Camastra S., Accili D., Ferrannini E. (2013). Human Insulin Resistance Is Associated with Increased Plasma Levels of 12α-Hydroxylated Bile Acids. Diabetes.

[199] Choucair I., Nemet I., Li L., Cole M.A., Skye S.M., Kirsop J.D., Fischbach M.A., Gogonea V., Brown J.M., Tang W.H.W. (2020). Quantification of bile acids: A mass spectrometry platform for studying gut microbe connection to metabolic diseases. J. Lipid Res..

[200] Sayin S.I., Wahlström A., Felin J., Jäntti S., Marschall H.U., Bamberg K., Angelin B., Hyötyläinen T., Orešič M., Bäckhed F. (2013). Gut microbiota regulates bile acid metabolism by reducing the levels of tauro-beta-muricholic acid, a naturally occurring FXR antagonist. Cell Metab..

[201] Hartman H.B., Gardell S.J., Petucci C.J., Wang S., Krueger J.A., Evans M.J. (2009). Activation of farnesoid X receptor prevents atherosclerotic lesion formation in LDLR-/- and apoE-/- mice. J. Lipid. Res..

[202] Mencarelli A., Renga B., Distrutti E., Fiorucci S. (2009). Antiatherosclerotic effect of farnesoid X receptor. Am. J. Physiol. Circ. Physiol..

[203] Watanabe M., Houten S., Mataki C., Christoffolete M., Kim B.W., Sato H., Messaddeq N., Harney J.W., Ezaki O., Kodama T. (2006). Bile acids induce energy expenditure by promoting intracellular thyroid hormone activation. Nature.

[204] Pols T.W., Nomura M., Harach T., Sasso G.L., Oosterveer M.H., Thomas C., Rizzo G., Gioiello A., Adorini L., Pellicciari R. (2011). TGR5 Activation Inhibits Atherosclerosis by Reducing Macrophage Inflammation and Lipid Loading. Cell Metab..

[205] Miyazaki-Anzai S., Masuda M., Levi M., Keenan A.L., Miyazaki M. (2014). Dual Activation of the Bile Acid Nuclear Receptor FXR and G-Protein-Coupled Receptor TGR5 Protects Mice against Atherosclerosis. PLoS ONE.

[206] Jadhav K., Xu Y., Xu Y., Li Y., Xu J., Zhu Y., Adorini L., Lee Y.K., Kasumov T., Yin L. (2018). Reversal of metabolic disorders by pharmacological activation of bile acid receptors TGR5 and FXR. Mol. Metab..

[207] Miyazaki-Anzai S., Masuda M., Kohno S., Levi M., Shiozaki Y., Keenan A.L., Miyazaki M. (2018). Simultaneous inhibition of FXR and TGR5 exacerbates atherosclerotic formation. J. Lipid Res..

[208] Tang W.H.W., Li D.Y., Hazen S.L. (2019). Dietary metabolism, the gut microbiome, and heart failure. Nat. Rev. Cardiol..

[209] Eblimit Z., Thevananther S., Karpen S.J., Taegtmeyer H., Moore D.D., Adorini L., Penny D.J., Desai M.S. (2018). TGR5 activation induces cytoprotective changes in the heart and improves myocardial adaptability to physiologic, inotropic, and pressure-induced stress in mice. Cardiovasc. Ther..

[210] Nagatomo Y., Tang W.H.W. (2015). Intersections Between Microbiome and Heart Failure: Revisiting the Gut Hypothesis. J. Card. Fail..

[211] Lekawanvijit S., Adrahtas A., Kelly D.J., Kompa A.R., Wang B.H., Krum H. (2010). Does indoxyl sulfate, a uraemic toxin, have direct effects on cardiac fibroblasts and myocytes?. Eur. Heart. J..

[212] Cason C.A., Dolan K.T., Sharma G., Tao M., Kulkarni R., Helenowski I.B., Doane B.M., Avram M.J., McDermott M.M., Chang E.B. (2018). Plasma microbiome-modulated indole- and phenyl-derived metabolites associate with advanced atherosclerosis and postoperative outcomes. J. Vasc. Surg..

[213] Lam V., Su J., Hsu A., Gross G.J., Salzman N.H., Baker J.E. (2016). Intestinal Microbial Metabolites Are Linked to Severity of Myocardial Infarction in Rats. PLoS ONE.

[214] Poesen R., Claes K., Evenepoel P., de Loor H., Augustijns P., Kuypers D.R., Meijers B. (2016). Microbiota-Derived Phenylacetylglutamine Associates with Overall Mortality and Cardiovascular Disease in Patients with CKD. J. Am. Soc. Nephrol..

[215] Nemet I., Saha P.P., Gupta N., Zhu W., Romano K.A., Skye S.M., Cajka T., Mohan M.L., Li L., Wu Y. (2020). A Cardiovascular Disease-Linked Gut Microbial Metabolite Acts via Adrenergic Receptors. Cell.

[216] Barnes D., Park K.T. (2017). Donor Considerations in Fecal Microbiota Transplantation. Curr. Gastroenterol. Rep..

[217] Fuentes S., de Vos W.M. (2016). How to Manipulate the Microbiota: Fecal Microbiota Transplantation. Adv. Exp. Med. Biol..

[218] Shreiner A.B., Kao J.Y., Young V.B. (2015). The gut microbiome in health and in disease. Curr. Opin. Gastroenterol..

[219] Ursell L.K., Metcalf J.L., Parfrey L.W., Knight R. (2012). Defining the human microbiome. Nutr. Rev..

[220] Gallo A., Passaro G., Gasbarrini A., Landolfi R., Montalto M. (2016). Modulation of microbiota as treatment for intestinal inflammatory disorders: An uptodate. World J. Gastroenterol..

[221] Cooper S., Mathews R., Bushar L., Paddock B., Wood J., Tammara R. (2019). The Human Microbiome: Composition and Change Reflecting Health and Disease. HAPS Educ..

[222] Vrieze A., Van Nood E., Holleman F., Salojärvi J., Kootte R.S., Bartelsman J.F., Dallinga-Thie G.M., Ackermans M.T., Serlie M.J., Oozeer R. (2012). Transfer of Intestinal Microbiota From Lean Donors Increases Insulin Sensitivity in Individuals With Metabolic Syndrome. Gastroenterology.

[223] Gregory J.C., Buffa J.A., Org E., Wang Z., Levison B.S., Zhu W., Wagner M.A., Bennett B.J., Li L., DiDonato J.A. (2015). Transmission of Atherosclerosis Susceptibility with Gut Microbial Transplantation. J. Biol. Chem..

[224] Proctor L.M. (2011). The Human Microbiome Project in 2011 and Beyond. Cell Host Microbe.

[225] Hu X.-F., Zhang W.-Y., Wen Q., Chen W.-J., Wang Z.-M., Chen J., Zhu F., Liu K., Cheng L.-X., Yang J. (2019). Fecal microbiota transplantation alleviates myocardial damage in myocarditis by restoring the microbiota composition. Pharmacol. Res..

[226] Drew L. (2016). Microbiota: Reseeding the gut. Nature.

[227] Brandt L.J. (2013). FMT: First step in a long journey. Am. J. Gastroenterol..

[228] De Leon L.M., Watson J.B., Kelly C.R. (2013). Transient Flare of Ulcerative Colitis After Fecal Microbiota Transplantation for Recurrent Clostridium difficile Infection. Clin. Gastroenterol. Hepatol..

[229] Chehoud C., Dryga A., Hwang Y., Nagy-Szakal D., Hollister E.B., Luna R.A., Versalovic J., Kellermayer R., Bushman F.D. (2016). Transfer of Viral Communities between Human Individuals during Fecal Microbiota Transplantation. mBio.

[230] Brand M.W., Wannemuehler M.J., Phillips G.J., Proctor A., Overstreet A.-M., Jergens A.E., Orcutt R.P., Fox J.G. (2015). The Altered Schaedler Flora: Continued Applications of a Defined Murine Microbial Community. ILAR J..

[231] Tamburini S., Shen N., Wu H.C., Clemente S.T.N.S.J.C. (2016). The microbiome in early life: Implications for health outcomes. Nat. Med..

[232] Hill C., Guarner F., Reid G., Gibson G.R., Merenstein D.J., Pot B., Morelli L., Canani R.B., Flint H.J., Salminen S. (2014). Expert consensus document: The International Scientific Association for Probiotics and Prebiotics consensus statement on the scope and appropriate use of the term probiotic. Nat. Rev. Gastroenterol. Hepatol..

[233] Yadav H., Jain S., Sinha P. (2007). Antidiabetic effect of probiotic dahi containing Lactobacillus acidophilus and Lactobacillus casei in high fructose fed rats. Nutrition.

[234] Tahri K., Grille J.P., Schneider F. (1996). Bifidobacteria Strain Behavior Toward Cholesterol: Coprecipitation with Bile Salts and Assimilation. Curr. Microbiol..

[235] Nguyen T., Kang J., Lee M. (2007). Characterization of Lactobacillus plantarum PH04, a potential probiotic bacterium with cholesterol-lowering effects. Int. J. Food Microbiol..

[236] Huang Y., Wang J., Quan G., Wang X., Yang L., Zhong L. (2014). Lactobacillus acidophilus ATCC 4356 Prevents Atherosclerosis via Inhibition of Intestinal Cholesterol Absorption in Apolipoprotein E-Knockout Mice. Appl. Environ. Microbiol..

[237] Asemi Z., Zare Z., Shakeri H., Sabihi S.-S., Esmaillzadeh A. (2013). Effect of Multispecies Probiotic Supplements on Metabolic Profiles, hs-CRP, and Oxidative Stress in Patients with Type 2 Diabetes. Ann. Nutr. Metab..

[238] Karlsson C., Ahrné S., Molin G., Berggren A., Palmquist I., Fredrikson G.N., Jeppsson B. (2010). Probiotic therapy to men with incipient arteriosclerosis initiates increased bacterial diversity in colon: A randomized controlled trial. Atherosclerosis.

[239] van Baarlen P., Troost F., van der Meer C., Hooiveld G., Boekschoten M., Brummer R.J.M., Kleerebezem M. (2010). Human mucosal in vivo transcriptome responses to three lactobacilli indicate how probiotics may modulate human cellular pathways. Proc. Natl. Acad. Sci. USA.

[240] Forslund K., Hildebrand F., Nielsen T., Falony G., Le Chatelier E., Sunagawa S., Prifti E., Vieira-Silva S., Gudmundsdottir V., Krogh Pedersen H. (2015). Disentangling type 2 diabetes and metformin treatment signatures in the human gut microbiota. Nature.

[241] Plovier H., Everard A., Druart C., Depommier C., Van Hul M., Geurts L., Chilloux J., Ottman N., Duparc T., Lichtenstein L. (2016). A purified membrane protein from Akkermansia muciniphila or the pasteurized bacterium improves metabolism in obese and diabetic mice. Nat. Med..

[242] Goodrich J.K., Waters J.L., Poole A.C., Sutter J.L., Koren O., Blekhman R., Beaumont M., Van Treuren W., Knight R., Bell J.T. (2014). Human Genetics Shape the Gut Microbiome. Cell.

[243] Gan X.T., Ettinger G., Huang C.X., Burton J.P., Haist J.V., Rajapurohitam V., Sidaway J.E., Martin G., Gloor G.B., Swann J.R. (2014). Probiotic Administration Attenuates Myocardial Hypertrophy and Heart Failure After Myocardial Infarction in the Rat. Circ. Heart Fail..

[244] Costanza A.C., Moscavitch S.D., Neto H.C.F., Mesquita E.T. (2015). Probiotic therapy with Saccharomyces boulardii for heart failure patients: A randomized, double-blind, placebo-controlled pilot trial. Int. J. Cardiol..

[245] Markowiak P., Śliżewska K. (2017). Effects of Probiotics, Prebiotics, and Synbiotics on Human Health. Nutrients.

[246] Kothari D., Patel S., Kim S.-K. (2019). Probiotic supplements might not be universally-effective and safe: A review. Biomed. Pharmacother..

[247] Khalesi S., Sun J., Buys N., Jayasinghe R. (2014). Effect of probiotics on blood pressure: A systematic review and meta-analysis of randomized, controlled trials. Hypertension.

[248] Xu Z., Knight R. (2015). Dietary effects on human gut microbiome diversity. Br. J. Nutr..

[249] Voreades N., Kozil A., Weir T.L. (2014). Diet and the development of the human intestinal microbiome. Front. Microbiol..

[250] Ravussin Y., Koren O., Spor A., LeDuc C., Gutman R., Stombaugh J., Knight R., Ley R.E., Leibel R.L. (2012). Responses of Gut Microbiota to Diet Composition and Weight Loss in Lean and Obese Mice. Obesity.

[251] David L.A., Maurice C.F., Carmody R.N., Gootenberg D.B., Button J.E., Wolfe B.E., Ling A.V., Devlin A.S., Varma Y., Fischbach M.A. (2014). Diet rapidly and reproducibly alters the human gut microbiome. Nature.

[252] Duncan S.H., Lobley G.E., Holtrop G., Ince J., Johnstone A.M., Louis P., Flint H.J. (2008). Human Colonic Microbiota Associated with Diet, Obesity and Weight Loss. Int. J. Obes..

[253] Duncan S.H., Belenguer A., Holtrop G., Johnstone A.M., Flint H.J., Lobley G.E. (2007). Reduced Dietary Intake of Carbohydrates by Obese Subjects Results in Decreased Concentrations of Butyrate and Butyrate-Producing Bacteria in Feces. Appl. Environ. Microbiol..

[254] Yang T., Aquino V., Lobaton G.O., Li H.B., Colon-Perez L., Goel R., Qi Y.F., Zubcevic J., Febo M., Richards E.M. (2019). Sustained Captopril-Induced Reduction in Blood Pressure Is Associated With Alterations in Gut-Brain Axis in the Spontaneously Hypertensive Rat. J. Am. Heart Assoc..

[255] Yisireyili M., Uchida Y., Yamamoto K., Nakayama T., Cheng X.W., Matsushita T., Nakamura S., Murohara T., Takeshita K. (2018). Angiotensin receptor blocker irbesartan reduces stress-induced intestinal inflammation via AT1a signaling and ACE2-dependent mechanism in mice. Brain, Behav. Immun..

[256] Wu D., Tang X., Ding L., Cui J., Wang P., Du X., Yin J., Wang W., Chen Y., Zhang T. (2019). Candesartan attenuates hypertension-associated pathophysiological alterations in the gut. Biomed. Pharmacother..

[257] Wu R., Mei X., Wang J., Sun W., Xue T., Lin C., Xu D. (2019). Zn(ii)-Curcumin supplementation alleviates gut dysbiosis and zinc dyshomeostasis during doxorubicin-induced cardiotoxicity in rats. Food Funct..

[258] Khan M.Y., Dirweesh A., Khurshid T., Siddiqui W.J. (2018). Comparing fecal microbiota transplantation to standard-of-care treatment for recurrent Clostridium difficile infection: A systematic review and meta-analysis. Eur. J. Gastroenterol. Hepatol..

[259] Khan T.J., Ahmed Y.M., Zamzami M.A., Mohamed S.A., Khan I., Baothman O.A.S., Mehanna M.G., Yasir M. (2018). Effect of atorvastatin on the gut microbiota of high fat diet-induced hypercholesterolemic rats. Sci. Rep..

[260] Epstein S., Speir E., Zhou Y., Guetta E., Leon M., Finkel T. (1996). The role of infection in restenosis and atherosclerosis: Focus on cytomegalovirus. Lancet.

[261] Patel P., Mendall M.A., Carrington D., Strachan D.P., Leatham E., Molineaux N., Levy J., Blakeston C., Seymour C.A., Camm A.J. (1995). Association of Helicobacter pylori and Chlamydia pneumoniae infections with coronary heart disease and cardiovascular risk factors. BMJ.

[262] Danesh J., Collins R., Peto R. (1997). Chronic infections and coronary heart disease: Is there a link?. Lancet.

[263] Saikku P., Mattila K., Nieminen M.S., Huttunen J.K., Leinonen M., Ekman M.R., Mäkelä P.H., Valtonen V. (1988). Serological Evidence of an Association of a Novel Chlamydia, Twar, with Chronic Coronary Heart Disease and Acute Myocardial Infarction. Lancet.

[264] Rune I., Rolin B., Larsen C., Nielsen D.S., Kanter J., Bornfeldt K.E., Lykkesfeldt J., Buschard K., Kirk R.K., Christoffersen B. (2016). Modulating the Gut Microbiota Improves Glucose Tolerance, Lipoprotein Profile and Atherosclerotic Plaque Development in ApoE-Deficient Mice. PLoS ONE.

[265] Galla S., Chakraborty S., Cheng X., Yeo J., Mell B., Zhang H., Mathew A.V., Vijay-Kumar M., Joe B. (2018). Disparate effects of antibiotics on hypertension. Physiol. Genom..

[266] Lam V., Su J., Koprowski S., Hsu A., Tweddell J.S., Rafiee P., Gross G.J., Salzman N.H., Baker J.E. (2012). Intestinal microbiota determine severity of myocardial infarction in rats. FASEB J..

[267] Santacruz A., Marcos A., Wärnberg J., Martí A., Martin-Matillas M., Campoy C., Moreno L.A., Veiga O., Redondo-Figuero C., Garagorri J.M. (2009). Interplay Between Weight Loss and Gut Microbiota Composition in Overweight Adolescents. Obesity.

[268] Mailing L.J., Allen J.M., Buford T.W., Fields C.J., Woods J.A. (2019). Exercise and the Gut Microbiome: A Review of the Evidence, Potential Mechanisms, and Implications for Human Health. Exerc. Sport Sci. Rev..

[269] Mohr A.E., Jäger R., Carpenter K.C., Kerksick C.M., Purpura M., Townsend J.R., West N.P., Black K., Gleeson M., Pyne D.B. (2020). The athletic gut microbiota. J. Int. Soc. Sports Nutr..

[270] Evans C.C., LePard K.J., Kwak J.W., Stancukas M.C., Laskowski S., Dougherty J., Moulton L., Glawe A., Wang Y., Leone V. (2014). Exercise Prevents Weight Gain and Alters the Gut Microbiota in a Mouse Model of High Fat Diet-Induced Obesity. PLoS ONE.

[271] Denou E., Marcinko K., Surette M.G., Steinberg G.R., Schertzer J.D. (2016). High-intensity exercise training increases the diversity and metabolic capacity of the mouse distal gut microbiota during diet-induced obesity. Am. J. Physiol. Endocrinol. Metab..

[272] Clarke S., Murphy E.F., O’Sullivan O., Lucey A., Humphreys M., Hogan A., Hayes P., O’Reilly M., Jeffery I., Wood-Martin R. (2014). Exercise and associated dietary extremes impact on gut microbial diversity. Gut.

[273] Aoki T., Oyanagi E., Watanabe C., Kobiki N., Miura S., Yokogawa Y., Kitamura H., Teramoto F., Kremenik M.J., Yano H. (2020). The Effect of Voluntary Exercise on Gut Microbiota in Partially Hydrolyzed Guar Gum Intake Mice under High-Fat Diet Feeding. Nutrients.

[274] Xiao S., Fei N., Pang X., Shen J., Wang L., Zhang B., Zhang M., Zhang X., Zhang C., Li M. (2014). A gut microbiota-targeted dietary intervention for amelioration of chronic inflammation underlying metabolic syndrome. FEMS Microbiol. Ecol..

[275] Shondelmyer K., Knight R., Sanivarapu A., Ogino S., Vanamala J.K.P. (2018). Ancient Thali Diet: Gut Microbiota, Immunity, and Health. Yale J. Biol. Med..

[276] Lira F.S., Rosa J.C., Pimentel G.D., Souza H.A., Caperuto E.C., CarnevaliJr L.C., Seelaender M., Damaso A.R., Oyama L.M., de Mello M.T. (2010). Endotoxin levels correlate positively with a sedentary lifestyle and negatively with highly trained subjects. Lipids Health Dis..

[277] Kallio K.A.E., Hätönen K.A., Lehto M., Salomaa V., Männistö S., Pussinen P.J. (2015). Endotoxemia, nutrition, and cardiometabolic disorders. Acta Diabetol..

[278] Liu Z., Liu H.-Y., Zhou H., Zhan Q., Lai W., Zeng Q., Ren H., Xu D. (2017). Moderate-Intensity Exercise Affects Gut Microbiome Composition and Influences Cardiac Function in Myocardial Infarction Mice. Front. Microbiol..

[279] Lustgarten M.S. (2019). The Role of the Gut Microbiome on Skeletal Muscle Mass and Physical Function: 2019 Update. Front. Physiol..

[280] Ortiz-Alvarez L., Xu H., Martinez-Tellez B. (2020). Influence of Exercise on the Human Gut Microbiota of Healthy Adults: A Systematic Review. Clin. Transl. Gastroenterol..

[281] Li J., Lin S., Vanhoutte P.M., Woo C.W., Xu A. (2016). Akkermansia Muciniphila Protects Against Atherosclerosis by Preventing Metabolic Endotoxemia-Induced Inflammation in Apoe-/- Mice. Circulation.

[282] Depommier C., Everard A., Druart C., Plovier H., Van Hul M., Vieira-Silva S., Falony G., Raes J., Maiter D., Delzenne N.M. (2019). Supplementation with Akkermansia muciniphila in overweight and obese human volunteers: A proof-of-concept exploratory study. Nat. Med..

[283] Ponziani F.R., Zocco M.A., D’Aversa F., Pompili M., Gasbarrini A. (2017). Eubiotic properties of rifaximin: Disruption of the traditional concepts in gut microbiota modulation. World J. Gastroenterol..

[284] Conraads V.M., Jorens P.G., De Clerck L.S., Van Saene H.K., Ieven M.M., Bosmans J.M., Schuerwegh A., Bridts C.H., Wuyts F., Stevens W.J. (2004). Selective intestinal decontamination in advanced chronic heart failure: A pilot trial. Eur. J. Heart Fail..

[285] Kumar S.A., Ward L.C., Brown L. (2016). Inulin oligofructose attenuates metabolic syndrome in high-carbohydrate, high-fat diet-fed rats. Br. J. Nutr..

[286] Alonso A., de la Fuente C., Martín-Arnau A.M., de Irala J., Martínez J.A., Martínez-González M. (2004). Fruit and vegetable consumption is inversely associated with blood pressure in a Mediterranean population with a high vegetable-fat intake: The Seguimiento Universidad de Navarra (SUN) Study. Br. J. Nutr..

[287] Sureda A., Del Mar Bibiloni M., Julibert A., Bouzas C., Argelich E., Llompart I., Pons A., Tur J.A. (2018). Adherence to the Mediterranean Diet and Inflammatory Markers. Nutrients.

[288] Saneei P., Hashemipour M., Kelishadi R., Esmaillzadeh A. (2014). The Dietary Approaches to Stop Hypertension (DASH) Diet Affects Inflammation in Childhood Metabolic Syndrome: A Randomized Cross-Over Clinical Trial. Ann. Nutr. Metab..

[289] Phillips C.M., Harrington J.M., Perry I.J. (2019). Relationship between dietary quality, determined by DASH score, and cardiometabolic health biomarkers: A cross-sectional analysis in adults. Clin. Nutr..

[290] Aguilar E.C., Leonel A.J., Teixeira L.G., Silva A.R., Silva J.F., Pelaez J.M.N., Capettini L.S.A., Lemos V.S., Santos R.A.S., Alvarez-Leite J.I. (2014). Butyrate impairs atherogenesis by reducing plaque inflammation and vulnerability and decreasing NFκB activation. Nutr. Metab. Cardiovasc. Dis..

[291] Aguilar E.C., dos Santos L.C., Leonel A.J., de Oliveira J.S., Santos E.A., Navia-Pelaez J.M., da Silva J.F., Mendes B.P., Capettini L.S., Teixeira L.G. (2016). Oral butyrate reduces oxidative stress in atherosclerotic lesion sites by a mechanism involving NADPH oxidase down-regulation in endothelial cells. J. Nutr. Biochem..

[292] Gibson G.R., Hutkins R., Sanders M.E., Prescott S.L., Reimer R.A., Salminen S.J., Scott K., Stanton C., Swanson K.S., Cani P.D. (2017). Expert consensus document: The International Scientific Association for Probiotics and Prebiotics (ISAPP) consensus statement on the definition and scope of prebiotics. Nat. Rev. Gastroenterol. Hepatol..

[293] De Filippis F., Pellegrini N., Vannini L., Jeffery I.B., La Storia A., Laghi L., Serrazanetti D.I., Di Cagno R., Ferrocino I., Lazzi C. (2016). High-level adherence to a Mediterranean diet beneficially impacts the gut microbiota and associated metabolome. Gut.

[294] Naqvi S., Asar T.O., Kumar V., Al-Abbasi F.A., Alhayyani S., Kamal M.A., Anwar F. (2021). A cross-talk between gut microbiome, salt and hypertension. Biomed. Pharmacother..

[295] Miura K., Greenland P., Stamler J., Liu K., Daviglus M.L., Nakagawa H. (2004). Relation of Vegetable, Fruit, and Meat Intake to 7-Year Blood Pressure Change in Middle-aged Men: The Chicago Western Electric Study. Am. J. Epidemiology.

[296] Bartolomaeus H., Balogh A., Yakoub M., Homann S., Markó L., Höges S., Tsvetkov D., Krannich A., Wundersitz S., Avery E.G. (2019). Short-Chain Fatty Acid Propionate Protects From Hypertensive Cardiovascular Damage. Circulation.

[297] Catry E., Bindels L.B., Tailleux A., Lestavel S., Neyrinck A.M., Goossens J.-F., Lobysheva I., Plovier H., Essaghir A., Demoulin J.-B. (2018). Targeting the Gut Microbiota with Inulin-Type Fructans: Preclinical Demonstration of a Novel Approach in the Management of Endothelial Dysfunction. Gut.

[298] Jin M., Qian Z., Yin J., Xu W., Zhou X. (2019). The role of intestinal microbiota in cardiovascular disease. J. Cell. Mol. Med..

[299] Lindskog Jonsson A., Caesar R., Akrami R., Reinhardt C., Fåk Hållenius F., Borén J., Bäckhed F. (2018). Impact of Gut Microbiota and Diet on the Development of Atherosclerosis in Apoe -/- Mice. Arterioscler. Thromb. Vasc. Biol..

[300] Korcz E., Kerényi Z., Varga L. (2018). Dietary fibers, prebiotics, and exopolysaccharides produced by lactic acid bacteria: Potential health benefits with special regard to cholesterol-lowering effects. Food Funct..

[301] Lew L.C., Choi S.B., Khoo B.Y., Sreenivasan S., Ong K.L., Liong M.T. (2018). Lactobacillus plantarum DR7 Reduces Cholesterol via Phosphorylation of AMPK That Down-regulated the mRNA Expression of HMG-CoA Reductase. Korean J. Food Sci. Anim. Resour..

[302] Ojetti V., Lauritano E.C., Barbaro F., Migneco A., Ainora M.E., Fontana L., Gabrielli M., Gasbarrini A. (2009). Rifaximin pharmacology and clinical implications. Expert Opin. Drug Metab. Toxicol..

[303] Delzenne N.M., Neyrinck A.M., Bäckhed F., Cani P.D. (2011). Targeting gut microbiota in obesity: Effects of prebiotics and probiotics. Nat. Rev. Endocrinol..

[304] Al Khodor S., Reichert B., Shatat I.F. (2017). The Microbiome and Blood Pressure: Can Microbes Regulate Our Blood Pressure?. Front Pediatr..

[305] A Petriz B., Castro A.P., Almeida J.A., Gomes C.P., Fernandes G.R., Kruger R.H., Pereira R.W., Franco O.L. (2014). Exercise induction of gut microbiota modifications in obese, non-obese and hypertensive rats. BMC Genom..

[306] Lambert J.E., Myslicki J.P., Bomhof M.R., Belke D.D., Shearer J., Reimer R.A. (2015). Exercise training modifies gut microbiota in normal and diabetic mice. Appl. Physiol. Nutr. Metab..

[307] Allen J.M., Mailing L.J., Niemiro G.M., Moore R., Cook M.D., White B.A., Holscher H.D., Woods J.A. (2018). Exercise Alters Gut Microbiota Composition and Function in Lean and Obese Humans. Med. Sci. Sports Exerc..

[308] Cerdá B., Pérez M., Pérez-Santiago J.D., Tornero-Aguilera J.F., González-Soltero R., Larrosa M. (2016). Gut microbiota modification: Another piece in the puzzle of the benefits of physical exercise in health?. Front Physiol..

[309] Yan Q., Zhai W., Yang C., Li Z., Mao L., Zhao M., Wu X. (2021). The Relationship among Physical Activity, Intestinal Flora, and Cardiovascular Disease. Cardiovasc. Ther..

[310] Pant K., Peixoto E., Richard S., Gradilone S.A. (2020). Role of Histone Deacetylases in Carcinogenesis: Potential Role in Cholangiocarcinoma. Cells.

[311] Ahmad A.F., Ward N., Dwivedi G. (2019). The gut microbiome and heart failure. Curr. Opin. Cardiol..

[312] Huang C.-C., Lin W.-T., Hsu F.-L., Tsai P.-W., Hou C.-C. (2009). Metabolomics investigation of exercise-modulated changes in metabolism in rat liver after exhaustive and endurance exercises. Eur. J. Appl. Physiol..

[313] Ganesh B., Nelson J.W., Eskew J.R., Ganesan A., Ajami N.J., Petrosino J.F., BryanJr R.M., Durgan D.J. (2018). Prebiotics, Probiotics, and Acetate Supplementation Prevent Hypertension in a Model of Obstructive Sleep Apnea. Hypertension.

[314] Ohira H., Tsutsui W., Fujioka Y. (2017). Are Short Chain Fatty Acids in Gut Microbiota Defensive Players for Inflammation and Atherosclerosis?. J. Atheroscler. Thromb..

[315] Vinolo M.A.R., Rodrigues H.G., Festuccia W.T., Crisma A.R., Alves V.S., Martins A.R., Amaral C.L., Fiamoncini J., Hirabara S.M., Sato F.T. (2012). Tributyrin attenuates obesity-associated inflammation and insulin resistance in high-fat-fed mice. Am. J. Physiol. Endocrinol. Metab..

[316] Papadaki A., Martinez-Gonzalez M.A., Alonso-Gómez A., Rekondo J., Salas-Salvadó J., Corella D., Ros E., Fitó M., Estruch R., Lapetra J. (2017). Mediterranean diet and risk of heart failure: Results from the PREDIMED randomized controlled trial. Eur. J. Heart Fail..

[317] Estruch R., Ros E., Salas-Salvadó J., Covas M.-I., Corella D., Arós F., Gómez-Gracia E., Ruiz-Gutiérrez V., Fiol M., Lapetra J. (2018). Primary Prevention of Cardiovascular Disease with a Mediterranean Diet Supplemented with Extra-Virgin Olive Oil or Nuts. N. Engl. J. Med..

[318] Zhu W., Wang Z., Tang W.H.W., Hazen S.L. (2017). Gut Microbe-Generated TMAO from Dietary Choline Is Prothrombotic in Subjects. Circulation.

[319] Li Q., Wu T., Liu R., Zhang M., Wang R. (2017). Soluble Dietary Fiber Reduces Trimethylamine Metabolism via Gut Microbiota and Co-Regulates Host AMPK Pathways. Mol. Nutr. Food Res..

[320] Hamaya R., Ivey K.L., Lee D.H., Wang M., Li J., Franke A., Sun Q., Rimm E.B. (2020). Association of diet with circulating trimethylamine-N-oxide concentration. Am. J. Clin. Nutr..

[321] Park J.E., Miller M., Rhyne J., Wang Z., Hazen S.L. (2019). Differential effect of short-term popular diets on TMAO and other cardio-metabolic risk markers. Nutr. Metab. Cardiovasc. Dis..

[322] Wu W.-K., Chen C.-C., Liu P.-Y., Panyod S., Liao B.-Y., Chen P.-C., Kao H.-L., Kuo H.-C., Kuo C.-H., Chiu T.H.T. (2019). Identification of TMAO-producer phenotype and host–diet–gut dysbiosis by carnitine challenge test in human and germ-free mice. Gut.

[323] Brunt V.E., Casso A.G., Gioscia-Ryan R.A., Sapinsley Z.J., Ziemba B.P., Clayton Z.S., Bazzoni A.E., VanDongen N.S., Richey J.J., Hutton D.A. (2021). Gut Microbiome-Derived Metabolite Trimethylamine N-Oxide Induces Aortic Stiffening and Increases Systolic Blood Pressure with Aging in Mice and Humans. Hypertension.

[324] Brunt V.E., Gioscia-Ryan R.A., Richey J.J., Zigler M.C., Cuevas L.M., Gonzalez A., Vázquez-Baeza Y., Battson M.L., Smithson A.T., Gilley A.D. (2019). Suppression of the gut microbiome ameliorates age-related arterial dysfunction and oxidative stress in mice. J. Physiol..

[325] Rogers M.A.M., Aronoff D.M. (2016). The influence of non-steroidal anti-inflammatory drugs on the gut microbiome. Clin. Microbiol. Infect..

[326] Brown J.M., Hazen S.L. (2017). Targeting of microbe-derived metabolites to improve human health: The next frontier for drug discovery. J. Biol. Chem..

[327] Craciun S., Marks J.A., Balskus E.P. (2014). Characterization of Choline Trimethylamine-Lyase Expands the Chemistry of Glycyl Radical Enzymes. ACS Chem. Biol..

[328] Xue J., Zhou D., Poulsen O., Imamura T., Hsiao Y.-H., Smith T.H., Malhotra A., Dorrestein P., Knight R., Haddad G.G. (2017). Intermittent Hypoxia and Hypercapnia Accelerate Atherosclerosis, Partially via Trimethylamine-Oxide. Am. J. Respir. Cell Mol. Biol..

[329] Chen K., Zheng X., Feng M., Li D., Zhang H. (2017). Gut Microbiota-Dependent Metabolite Trimethylamine N-Oxide Contributes to Cardiac Dysfunction in Western Diet-Induced Obese Mice. Front. Physiol..

[330] Roberts A., Gu X., Buffa J.A., Hurd A.G., Wang Z., Zhu W., Gupta N., Skye S.M., Cody D.B., Levison B.S. (2018). Development of a gut microbe–targeted nonlethal therapeutic to inhibit thrombosis potential. Nat. Med..

[331] Cashman J.R., Xiong Y., Lin J., Verhagen H., van Poppel G., van Bladeren P.J., Larsen-Su S., Williams D.E. (1999). In vitro and in vivo inhibition of human flavin-containing monooxygenase form 3 (FMO3) in the presence of dietary indoles. Biochem. Pharmacol..

[332] Mitchell S.C., Smith R.L. (2001). Trimethylaminuria: The fish malodor syndrome. Drug Metab. Dispos..

[333] Velasquez M.T., Ramezani A., Manal A., Raj D.S. (2016). Trimethylamine N-Oxide: The Good, the Bad and the Unknown. Toxins.

[334] Chittim C.L., Del Campo A.M., Balskus E.P. (2019). Gut bacterial phospholipase Ds support disease-associated metabolism by generating choline. Nat. Microbiol..

[335] Chen Y.-S., Liu H.-M., Lee T.-Y. (2019). Ursodeoxycholic Acid Regulates Hepatic Energy Homeostasis and White Adipose Tissue Macrophages Polarization in Leptin-Deficiency Obese Mice. Cells.

[336] Úbeda M., Lario M., Muñoz L., Borrero M.J., Rodríguez-Serrano M., Sánchez-Díaz A.M., Del Campo R., Lledó L., Pastor Ó., García-Bermejo L. (2016). Obeticholic acid reduces bacterial translocation and inhibits intestinal inflammation in cirrhotic rats. J. Hepatol..

[337] Pellicciari R., Costantino G., Camaioni E., Sadeghpour B.M., Entrena A., Willson T.M., Fiorucci S., Clerici C., Gioiello A. (2004). Bile acid derivatives as ligands of the farnesoid X receptor. Synthesis, evaluation, and structure-activity relationship of a series of body and side chain modified analogues of chenodeoxycholic acid. J. Med. Chem..

[338] Joyce S.A., MacSharry J., Casey P.G., Kinsella M., Murphy E.F., Shanahan F., Hill C., Gahan C.G.M. (2014). Regulation of host weight gain and lipid metabolism by bacterial bile acid modification in the gut. Proc. Natl. Acad. Sci. USA.

[339] Korpela K., Salonen A., Virta L.J., Kekkonen R.A., Forslund K., Bork P., De Vos W.M. (2016). Intestinal microbiome is related to lifetime antibiotic use in Finnish pre-school children. Nat. Commun..

[340] Caparrós-Martín J.A., Lareu R.R., Ramsay J.P., Peplies J., Reen F.J., Headlam H.A., Ward N.C., Croft K.D., Newsholme P., Hughes J.D. (2017). Statin Therapy Causes Gut Dysbiosis in Mice through a PXR-Dependent Mechanism. Microbiome.

[341] Williams B.B., Van Benschoten A.H., Cimermancic P., Donia M.S., Zimmermann M., Taketani M., Ishihara A., Kashyap P.C., Fraser J.S., Fischbach M.A. (2014). Discovery and Characterization of Gut Microbiota Decarboxylases that Can Produce the Neurotransmitter Tryptamine. Cell Host Microbe.

[342] Pott A.S., Dahl C. (1998). Sirohaem sulfite reductase and other proteins encoded by genes at the dsr locus of Chromatium vinosum are involved in the oxidation of intracellular sulfur. Microbiology.

[343] London J., Goldberg M.E. (1972). The Tryptophanase from Escherichia coli K-12: I. Purification, Physical Properties, and Quaternary Structure. J. Biol. Chem..

[344] Peck S.C., Denger K., Burrichter A., Irwin S.M., Balskus E.P., Schleheck D. (2019). A glycyl radical enzyme enables hydrogen sulfide production by the human intestinal bacterium *Bilophila wadsworthia*. Proc. Natl. Acad. Sci. USA.

[345] Lavelle A., Lennon G., O’Sullivan O., Docherty N., Balfe A., Maguire A., Mulcahy H.E., Doherty G., O’Donoghue D., Hyland J. (2015). Spatial variation of the colonic microbiota in patients with ulcerative colitis and control volunteers. Gut.

[346] Salonen A., Lahti L., Salojärvi J., Holtrop G., Korpela K., Duncan S.H., Date P., Farquharson F., Johnstone A.M., Lobley G.E. (2014). Data from: Impact of diet and individual variation on intestinal microbiota composition and fermentation products in obese men. ISME J..

[347] Kuntz T.M., Gilbert J.A. (2017). Introducing the Microbiome into Precision Medicine. Trends Pharmacol. Sci..

